# Regulation of tau internalization, degradation, and seeding by LRP1 reveals multiple pathways for tau catabolism

**DOI:** 10.1016/j.jbc.2021.100715

**Published:** 2021-04-28

**Authors:** Joanna M. Cooper, Aurelien Lathuiliere, Mary Migliorini, Allison L. Arai, Mashhood M. Wani, Simon Dujardin, Selen C. Muratoglu, Bradley T. Hyman, Dudley K. Strickland

**Affiliations:** 1 The Center for Vascular and Inflammatory Diseases, University of Maryland School of Medicine, Baltimore, Maryland, USA; 2 Alzheimer Research Unit, Department of Neurology, Massachusetts General Hospital and Harvard Medical School, Charlestown, Massachusetts, USA; 3 Department of Physiology, University of Maryland School of Medicine, Baltimore, Maryland, USA; 4 Department of Surgery, University of Maryland School of Medicine, Baltimore, Maryland, USA

**Keywords:** LRP1, tau, Alzheimer' s disease, catabolism, Apolipoprotien E, neurofibrillary tangles, surface plasmon resonance, tau spreading, lipoprotein receptors, neurons, AD, Alzheimer's disease, apoE, apolipoprotein E, APP, amyloid precursor protein, ATCC, American Type Culture Collection, CHO, Chinese hamster ovary, CR, complement-like repeat, CQ, chloroquine, DMEM, Dulbecco's modified Eagle' medium, FBS, fetal bovine serum, HEK293T, human embryonic kidney 293T, HMW, high–molecular weight, HSPG, heparan sulfate proteoglycan, LDL, low-density lipoprotein, LRP1, low-density lipoprotein receptor–related protein 1, MEF, mouse embryonic fibroblast, NFT, neurofibrillary tangle, RAP, receptor-associated protein, SEC, seeding-competent, SPR, surface plasmon resonance

## Abstract

In Alzheimer's disease (AD), pathological forms of tau are transferred from cell to cell and “seed” aggregation of cytoplasmic tau. Phosphorylation of tau plays a key role in neurodegenerative tauopathies. In addition, apolipoprotein E (apoE), a major component of lipoproteins in the brain, is a genetic risk determinant for AD. The identification of the apoE receptor, low-density lipoprotein receptor–related protein 1 (LRP1), as an endocytic receptor for tau raises several questions about the role of LRP1 in tauopathies: is internalized tau, like other LRP1 ligands, delivered to lysosomes for degradation, and does LRP1 internalize pathological tau leading to cytosolic seeding? We found that LRP1 rapidly internalizes ^125^I-labeled tau, which is then efficiently degraded in lysosomal compartments. Surface plasmon resonance experiments confirm high affinity binding of tau and the tau microtubule-binding domain to LRP1. Interestingly, phosphorylated forms of recombinant tau bind weakly to LRP1 and are less efficiently internalized by LRP1. LRP1-mediated uptake of tau is inhibited by apoE, with the apoE4 isoform being the most potent inhibitor, likely because of its higher affinity for LRP1. Employing post-translationally–modified tau derived from brain lysates of human AD brain tissue, we found that LRP1-expressing cells, but not LRP1-deficient cells, promote cytosolic tau seeding in a process enhanced by apoE. These studies identify LRP1 as an endocytic receptor that binds and processes monomeric forms of tau leading to its degradation and promotes seeding by pathological forms of tau. The balance of these processes may be fundamental to the spread of neuropathology across the brain in AD.

In Alzheimer's disease (AD), neurofibrillary tangles (NFTs) have a sequential accumulation pattern as the disease progresses that correlates with neuronal susceptibility and cognitive decline (1, 2, 3). NFTs consist of abnormal accumulations of excessively phosphorylated forms of the microtubule-associated protein tau within the cytoplasm of certain neurons. In mouse models of AD in which human mutant P301L tau is overexpressed in the entorhinal cortex, aggregated tau accumulates in brain regions with neuronal projections from the entorhinal cortex such as the dentate gyrus, supporting the notion that the pathological tau protein can spread from one nonadjacent anatomical region of the brain to another (4, 5, 6, 7). In this process, pathological forms of tau are thought to be transferred from cell to cell and “seed” aggregation of cytoplasmic tau by a prion-like templated misfolding of endogenous tau (8, 9, 10, 11, 12).

Mechanisms of tau spreading are not well understood, but the presence of extracellular tau in brain interstitial fluid (13) led to the discovery that tau is constitutively secreted from neurons in a manner that is increased during neuronal activity and upon aging (14, 15, 16, 17, 18). Extracellular tau aggregates can transfer between cocultured cells, are internalized by cells, and following endocytosis can in some instances induce fibrillization of intracellular tau (19, 20). Recent studies have provided evidence that the low-density lipoprotein (LDL) receptor–related protein 1 (LRP1) functions as an endocytic neuronal receptor for the uptake and spread of tau (21).

LRP1 is a large endocytic and signaling receptor that binds numerous ligands and effectively delivers them to endosomal compartments where they dissociate and are delivered to lysosomal compartments for degradation. LRP1 modulates the trafficking of transmembrane receptors, including amyloid precursor protein (APP) (22, 23, 24). The association of APP with LRP1 leads to enhanced amyloidogenic processing of APP increasing production of the Aβ (23, 25, 26). In addition, LRP1 directly binds Aβ and mediates its clearance from the brain at the blood brain barrier (27, 28, 29) suggesting a dual contribution of LRP1 to AD. LRP1 is highly expressed throughout the brain in neurons, astrocytes, microvascular endothelial cells, and microglia and is a major apolipoprotein E (apoE) receptor. ApoE genotype has a strong impact on development of late-onset AD, with the ε4 allele representing a risk factor and the ε2 allele being protective (30, 31). Thus, it is especially interesting that LRP1 also plays a role in tau uptake as well, providing an unusual confluence of molecules implicated in Alzheimer pathophysiology in a single molecular pathway.

However, the simple explanation that LRP1 delivers tau to the cytoplasm for seeded misfolding of endogenous tau is complicated by the observation that nearly all other LRP1 ligands are delivered to lysosomes for degradation. Further, it is not known if LRP1 preferentially mediates uptake of physiological tau or of the heavily post-translationally–modified tau that participates in the tau seeding in the cytoplasm. The current study employed well-characterized cell lines containing, or deficient in, LRP1 to address these questions. Our studies reveal that LRP1 efficiently mediates delivery of recombinant tau to lysosomal compartments for degradation. By contrast, cells expressing LRP1, but not cells deficient in LRP1, also evidence endolysosomal escape of human brain–derived post-translationally–modified tau and promote tau seeding in the cytoplasm, suggesting the possibility of differential LRP1-mediated trafficking of tau based on its conformational or charge characteristics.

## Results

### LRP1-mediated endocytosis of recombinant tau results in lysosomal degradation of tau

**Figure 1:**
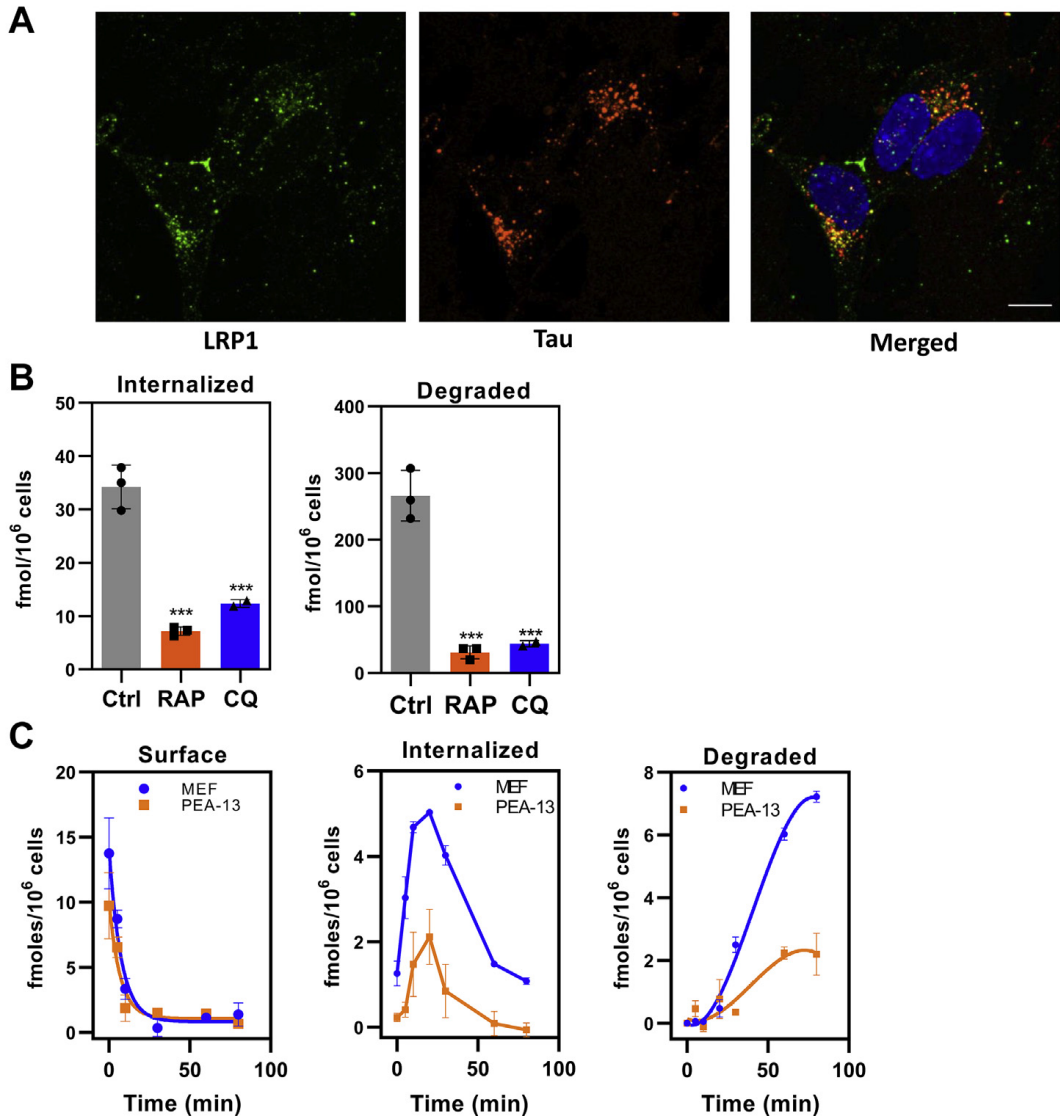
**Tau is efficiently degraded following LRP1-mediated internalization.***A*, human neuroblastoma cells (SH-SY5Y) were incubated with monoclonal antibody 5A6 conjugated with Alexa Fluor 488 (*green*) to label the endocytic pool of LRP1, followed by 20 nM tau conjugated with Alexa Fluor 594 (*red*). The cells were then fixed and imaged. Colocalization of functional LRP1 and internalized tau is displayed on merged panel (*yellow*). The scale bar represents 10 μm. *B*, WI-38 cells were incubated with 20 nM ^125^I-labeled tau in the absence or the presence of 1 μM RAP for 2 h at 37 °C, and the steady-state levels internalized (*right panel*) and degraded (*left panel*) quantified. Internalization and degradation were also measured in the presence or the absence of 100 μM chloroquine (CQ) (shown are means ± SEM; one-way ANOVA followed by Tukey's multiple comparisons test) (∗∗∗*p* < 0.001 compared with control, n = 3). *C*, MEF cells or LRP1-deficient PEA-13 cells were incubated with 20 nM ^125^I-labeled tau at 4 °C for 2 h in the presence or the absence of 1 μM RAP, then the media were replaced with warm assay media, and cells were incubated at 37 °C for specified times. The amount of RAP-sensitive surface bound, internalized, and degraded ^125^I-labeled tau was quantified (shown are means ± SEM, n = 3). LRP1, low-density lipoprotein receptor–related protein 1; MEF, mouse embryonic fibroblast; RAP, receptor-associated protein.

Alternative splicing of the *MAPT* gene gives rise to six variants of tau protein, with the 2N4R variant being the largest. In all our experiments, the 2N4R variant was used unless otherwise noted. When we examined the internalization of tau in the neuroblastoma cell line SH-SY5Y with functional LRP1 that is fluorescently labeled (32), we found that tau colocalized with LRP1 within punctate structures resembling endosomal/lysosomal compartments (Fig. 1*A*).

We next investigated the internalization and degradation of ^125^I-labeled tau in WI-38 fibroblasts, which express high levels of LRP1 and efficiently degrade LRP1 ligands. These experiments revealed that internalized tau is effectively degraded in a process that is inhibited by the LRP1 antagonist, receptor-associated protein (RAP) (33). This degradation occurs in lysosomal compartments as demonstrated by the ability of the lysosomal inhibitor, chloroquine (CQ), to block tau degradation (Fig. 1*B*). Curiously, CQ also reduced the amount of measured tau internalized (Fig. 1*A*). In addition to inhibiting lysosome acidification thereby reducing the activity of lysosomal proteases, CQ also inhibits endosomal acidification, and we hypothesize that this causes inefficient dissociation of tau from LRP1 possibly resulting in recycling of the LRP1–tau complex back to the cell surface, thereby limiting the amount of tau internalized.

To examine the cellular processing of tau mediated by LRP1, we performed a single-cycle endocytosis experiment, in which ^125^I-labeled tau was first incubated with cells at 4 °C for 1 h to prevent endocytosis. For this experiment, we used mouse embryonic fibroblasts (MEFs) expressing LRP1 or lacking LRP1 (PEA-13) and incubated tau in the presence or the absence of RAP to block LRP1-mediated binding. After washing, the media was replaced with fresh media at 37 °C in the absence or the presence of RAP to trigger endocytosis, and the amount of tau on the cell surface, internalized, or degraded, was quantified. In both cell types, a rapid disappearance of tau from the cell surface occurred with a half-life of ∼5 min (Fig. 1*C*, *left panel*). Some of the tau dissociated from the cell surface into the media, but most was internalized into the cells (Fig. 1*C*, *middle panel*). After a lag period of approximately 15 min, tau degradation was detected (Fig. 1*C*, *right panel*). Tau surface binding, internalization, and degradation were reduced, but not absent, in the LRP1-deficient PEA-13 cells. These data reveal that LRP1-mediated endocytosis results in effective trafficking of tau to lysosomal compartments and subsequent degradation. The results also confirm that LRP1-deficient fibroblasts internalize and degrade tau, although at reduced levels.

### Uptake of ^125^I-labeled tau in LRP1-deficient Chinese hamster ovary cells confirms the existence of additional receptor mechanisms for tau uptake

**Figure 2:**
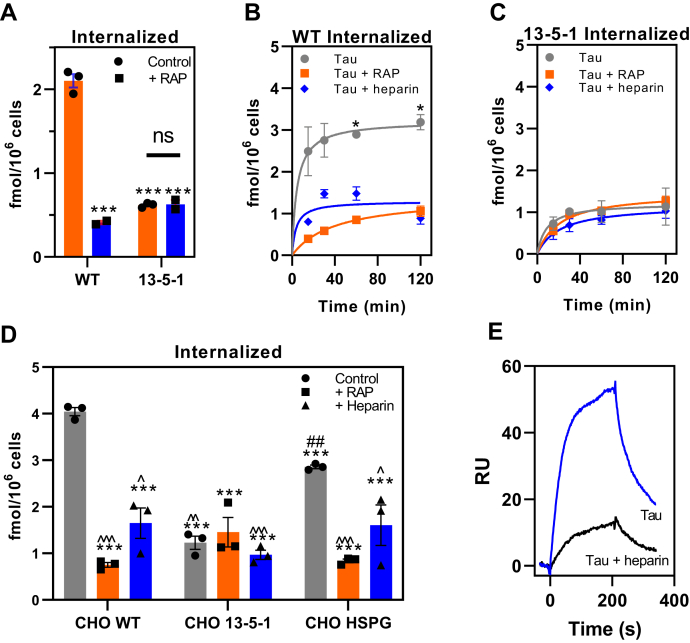
**Uptake of tau in LRP1-deficient CHO cells confirms the existence of additional receptors for tau uptake.***A*, steady-state levels of ^125^I-labeled tau (20 nM) internalized in WT or LRP1-deficient 13-5-1 CHO cells when incubated in the absence or the presence of 1 μM RAP for 2 h at 37 °C. *B* and *C*, time course for internalization of ^125^I-labeled tau (20 nM) in CHO WT (*B*) and CHO 13-5-1 (*C*) cells in the presence or the absence of RAP (1 mM) or heparin (20 mg/ml). *D*, WT, 13-5-1 and HSPG-deficient (CHO HSPG) CHO cells were incubated with 20 nM ^125^I-labeled tau in the absence or the presence of RAP (1 mM) or heparin (20 mg/ml) at 37 °C for 2 h, and internalized tau was measured. *E*, SPR analysis of 10 nM tau binding to LRP1 in the absence or the presence of 20 mg/ml heparin. *A*–*D*, means ± SEM; two-way ANOVA followed by Sidak's multiple comparisons test, (*A*) ∗∗∗*p* < 0.0001 compared with WT control, n = 3; (*B* and *C*) ∗*p* < 0.0001 comparison of tau *versus* tau + RAP, n = 3; (*D*) significance reported compared with ∗CHO WT, #CHO 13-5-1, or ˆCHO HSPG (one symbol *p* < 0.03; two symbols *p* < 0.007; and three symbols *p* < 0.0001). CHO, Chinese hamster ovary; HSPG, heparan sulfate proteoglycan; LRP1, low-density lipoprotein receptor–related protein 1; RAP, receptor-associated protein; SPR, surface plasmon resonance.

The presence of residual uptake and degradation of tau in LRP1-deficient cells suggested the existence of LRP1-independent mechanism(s) for tau internalization. We further examined this idea by studying the endocytosis of ^125^I-labeled tau in WT Chinese hamster ovary (CHO) cells and CHO 13-5-1 cells, the latter of which are deficient in LRP1 (34). The results reveal that the cellular uptake of ^125^I-labeled tau was significantly reduced, by about two thirds, in CHO cells lacking LRP1 (Fig. 2*A*). The contribution of LRP1 to cellular-mediated uptake of tau was further confirmed by demonstrating that RAP largely prevented the uptake of tau in WT CHO cells. When we examined the time course of tau internalization, we observed that CHO cells rapidly internalize ^125^I-labeled tau with a *t*_1/2_ of ∼5 min in a process that is reduced by both RAP and heparin (Fig. 2*B*), which has previously been reported to block tau internalization (35). The fact that CHO 13-5-1 cells appear to internalize small amounts of ^125^I-labeled tau that is not inhibited by either RAP or heparin (Fig. 2*C*) confirms the existence of LRP1-independent pathways for tau internalization in these cells, which accounts for approximately 30% of tau internalization.

Previous studies have suggested that heparan sulfate proteoglycans (HSPGs) regulate the cellular uptake of tau (35, 36, 37). Thus, we also examined the uptake of ^125^I-labeled tau in CHO cells deficient in xylosyltransferase (38), an enzyme that catalyzes the first step in glycosaminoglycan synthesis. These cells lack HSPG as well as the glycosaminoglycans such as chondroitin sulfate and heparan sulfate. Our experiment compared the extent of ^125^I-labeled tau internalized these cells (labeled CHO HSPG) along with WT and LRP1-deficient CHO cells. The results of this experiment (Fig. 2*D*) show a significant reduction in the amount of ^125^I-labeled tau internalized in HSPG-deficient CHO cells when compared with WT CHO cells. RAP reduced the uptake of ^125^I-labeled tau in HSPG-deficient CHO cells, but our initial experiment has no significant effect on ^125^I-labeled tau uptake in LRP1-deficient CHO cells. These results suggest that glycosaminoglycans participate in the LRP1-mediated uptake of tau, similar to what we have observed for LRP1-mediated very low-density lipoprotein uptake induced by lipoprotein lipase (39). The fact that heparin reduces uptake in the CHO HSPG cells is consistent with our binding studies revealing that heparin inhibits the binding of tau to LRP1 (Fig. 2*E*). Although LRP1 blockade with RAP in HSPG-deficient CHO cells reduced levels of tau uptake, it would be ideal to genetically eliminate both LRP1 and HSPG to absolutely confirm if other receptors are involved in tau uptake. In the case of Aß aggregates, uptake can be mediated by a wide range of receptors (40), and the same may be true for tau.

### What forms of tau are recognized by LRP1?

Tau contains two major domains: an N-terminal “projection” domain containing the alternatively spliced N1 and N2 regions and the C-terminal microtubule-binding domain (MBD) containing four highly conserved repeat regions, R1–R4, which binds to microtubules (41). In addition, tau contains multiple serine, threonine, and tyrosine phosphorylation sites that have been extensively studied as phosphorylation is a common post-translational modification of tau (42, 43, 44) that are detected in tau aggregates in AD and other tauopathies (12, 45, 46, 47).

**Figure 3:**
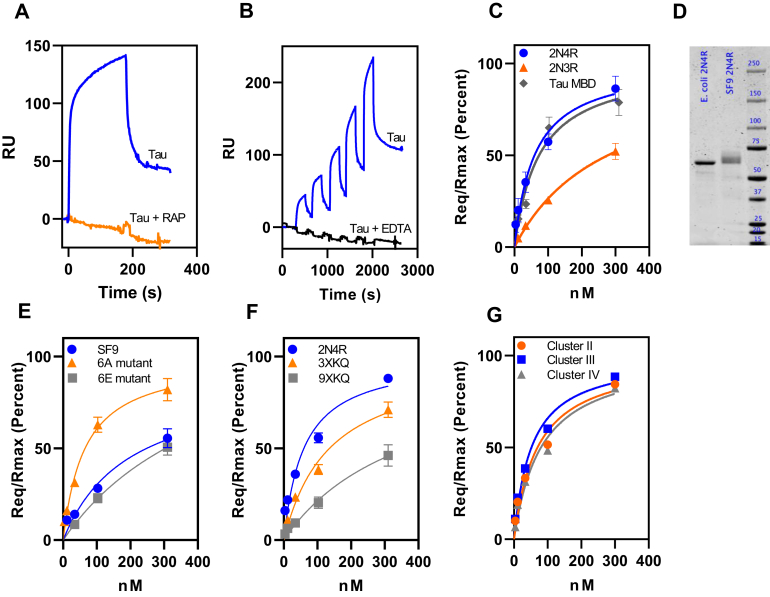
**Phosphorylated forms of tau bind weakly to LRP1.***A*, inhibition of tau binding to LRP1 by excess RAP as assessed by coinjection experiment. *B*, single-cycle kinetic experiment quantifying binding of monomeric tau (3.8, 11.5, 34.4, 103.3, and 310 nM) to LRP1 in the presence of Ca^2+^ (*blue line*) or EDTA (*black line*). *C*, binding of tau isoforms 2N4R, 2N3R, and tau MBD to LRP1 assessed by SPR equilibrium analysis. *D*, about 1 μg of recombinant tau produced in *Escherichia coli* or SF9 cells was ran on a 4 to 12% gel and stained with colloidal Coomassie. Image was captured using Licor. Quantification of bands reveals a signal of 2270 for *E. coli* tau and 2280 for SF9 tau. *E*, the binding of tau produced by Sf9 cells along with two mutant forms of tau to full-length human LRP1 was measured by SPR; 6A (T181, S199, S202, S396, S400, and S404 are all converted to alanine) and 6E, in which all these residues are converted to glutamic acid. *F*, binding of mutant forms of tau to LRP1: 3XKQ in which lysine residues 311, 317, and 321 were converted to glutamine residues and 9XKQ tau in which lysine residues 311, 217, 321, 340, 343, 347, 353, 369, and 375 are all converted to glutamine residues. *G*, binding of monomeric tau to LRP1 clusters II, III, or IV by SPR equilibrium analysis. For all experiments, *n* = 3 (biological replicates), (*A* and *B*) show representative data, (*C*, *E*, and *F*) show means ± SEM. LRP1, low-density lipoprotein receptor–related protein 1; MBD, microtubule-binding domain; RAP, receptor-associated protein; SPR, surface plasmon resonance.

To quantify the interaction of various forms of tau with LRP1, we employed surface plasmon resonance (SPR) experiments. To confirm the specificity of the interaction of tau with coupled LRP1, we demonstrated that RAP blocks the binding of tau to the LRP1-coated SPR surfaces (Fig. 3*A*). For subsequent experiments, we used single-cycle kinetic experiments to quantify the binding of tau to LRP1. We noted that the binding of tau to the LRP1-coated chip was ablated in the presence of EDTA, which chelates the essential Ca^2+^ ions necessary to stabilize the LDLa ligand-binding repeats, which are critical for ligand binding by this class of receptors (Fig. 3*B*, *black lines*) further confirming the specificity of the interaction. To determine the *K*_*D*_ of this interaction, we fit the individual data to a pseudo first-order process to obtain values of Req for each concentration of tau isoforms (2N4R, 2N3R, and tau MBD) and then plotted the Req values as a function of total concentration of tau (Fig. 3*C*). Nonlinear regression analysis of the plot revealed a *K*_*D*_ value of 60 ± 8 nM for the 2N4R tau isoform, a value comparable to other LRP1 ligands such as soluble forms of APP (22) or hepatic lipase (48).

Interestingly, the 2N3R isoform of tau that lacks the second microtubule-binding repeat (R2) encoded by the alternatively spliced exon 10, bound to LRP1 with considerably weaker affinity (*K*_*D*_ = 278 ± 55 nM; Fig. 3*C*) suggesting that the R2 domain of tau contributes to the interaction of tau with LRP1. We also quantified the interaction of the MBD (R1–R4, leu243–glu372) with LRP1 using SPR measurements, and the results of these experiments reveal that this region of tau binds to LRP1 with an affinity similar to the intact molecule (*K*_*D*_ = 73 ± 18 nM) (Fig. 3*C*). These results indicate that the MBD alone is sufficient for binding to LRP1.

We also tested the hypothesis that phosphorylation of tau might alter its binding to LRP1 by examining the binding of recombinant tau produced by Sf9 insect cells, which produce well-characterized hyperphosphorylated forms of tau (49, 50). SDS-PAGE analysis confirmed a slower migration for the hyperphosphorylated form as expected (Fig. 3*D*). We found that hyperphosphorylated tau produced by Sf9 cells bound LRP1 with a fourfold weaker affinity (*K*_*D*_ = 243 ± 17 nM) (Fig. 3*E*). We also examined the binding of two recombinant (*Escherichia coli* produced) mutant forms of tau to LRP1: mutant 6A, in which T181, S199, S202, S396, S400, and S404 are all converted to alanine, and mutant 6E, in which all these residues are converted to the phosphomimetic glutamic acid. These specific residues have been found to be phosphorylated to a greater extent in AD brains (51). Our results reveal that the 6A mutant binds to LRP1 with a *K*_*D*_ value similar to that of WT tau (*K*_*D*_ = 65 ± 4 nM), whereas the 6E mutant binds to LRP1 with fivefold weaker affinity (*K*_*D*_ = 321 ± 17 nM) (Fig. 3*E*). Together, the results of our studies reveal that phosphorylated forms of tau bind to LRP1 with significantly lower affinity.

Prior studies have confirmed the importance of lysine residues on ligands that are critical for the ligand–LRP1 interaction (52, 53, 54), and modifying lysines on the tau K18 MBD peptide with sulfo-*N*-hydroxysulfosuccinimide acetate prevented uptake in H4 cells (21). To identify specific lysine residues that may be involved in interaction with LRP1, we examined the binding of mutated forms of tau in which lysine residues 311, 317, and 321 were all converted to glutamine residues (3XKQ) and a form of tau in which lysine residues 311, 217, 321, 340, 343, 347, 353, 369, and 375 are all converted to glutamine residues (9XKQ). SPR analysis revealed that the 3XKQ form of tau bound to LRP1 with a twofold weaker affinity (*K*_*D*_ = 136 ± 6 nM), whereas the 9XKQ form of tau bound to LRP1 with a sixfold reduction in affinity (*K*_*D*_ = 372 ± 33 nM) revealing an important role for these lysine residues in binding to LRP1 (Fig. 3*F*).

The ligand-binding regions of LRP1 are mainly localized to clusters of LDLa repeats, termed clusters I, II, III, and IV. To determine which region of LRP1 is involved in tau binding, we investigated the binding of tau to clusters II, III, and IV immobilized on SPR chips. The results of a single-cycle kinetic experiment confirm that tau readily binds to clusters II, III, and IV with *K*_*D*_ values of 69 ± 25, 52 ± 14, and 81 ± 29 nM, respectively (Fig. 3*G*). The binding of tau to all three clusters of LRP1 with similar affinity is unusual as most ligands prefer to bind to clusters II or IV (55). Our results differ from those of Rauch *et al.* (21) who found that LRP1 minireceptors containing clusters II and IV could both mediate tau internalization but not those minireceptors expressing cluster III. Possibly the difference in results arise from the fact that mini-LRP1 expressing cluster III is not effectively processed in cells (56).

**Figure 4:**
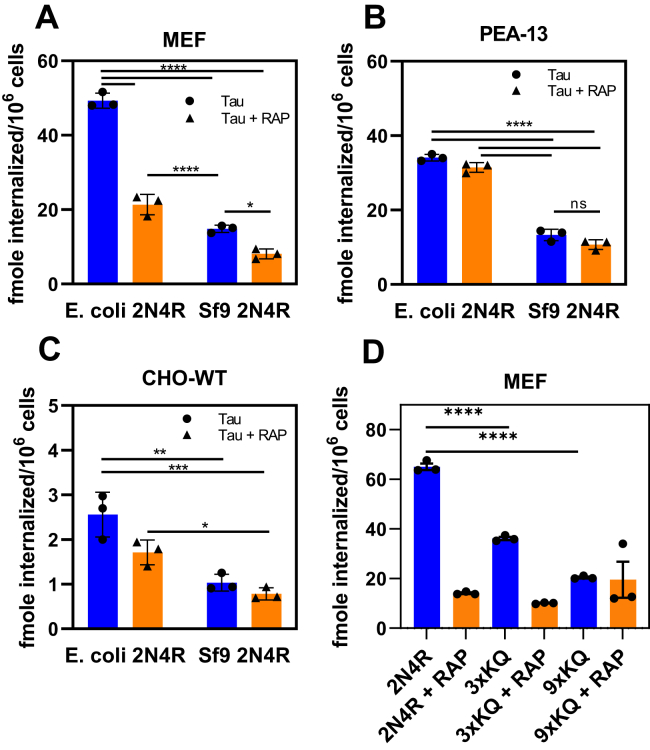
**Hyperphosphorylated forms of tau are not efficiently internalized by LRP1.** Internalization of 20 nM ^125^I-labeled WT tau or Sf9-produced tau in MEF cells (*A*) LRP1-deficient PEA-13 cells (*B*) or CHO-WT cells (*C*) in the presence or the absence of RAP; *D*, internalization of 20 nM of ^125^I-labeled WT tau, 3XKQ, or 9XKQ mutants. (Two-way ANOVA, Sidak's multiple comparisons test; ∗∗∗∗*p* < 0.0001, ∗∗∗*p* < 0.0007, ∗∗*p* < 0.002, ∗*p* < 0.04, n = 3). CHO, Chinese hamster ovary; LRP1, low-density lipoprotein receptor–related protein 1; MEF, mouse embryonic fibroblast; RAP, receptor-associated protein.

To determine if the LRP1-mediated uptake of post-translationally–modified forms of tau is also diminished, we examined the internalization of recombinant phosphorylated tau produced by Sf9 insect cells compared with tau without any post-translational modifications produced by *E. coli.* In this experiment, 20 nM of ^125^I-labeled tau produced in *E. coli* or in Sf9 insect cells was incubated with LRP1-expressing MEF or CHO WT cells along with LRP1-deficient PEA-13 fibroblasts for 2 h at 37 °C, and the amount of tau internalized quantified. The results of this experiment reveal that hyperphosphorylated forms of tau are less efficiently internalized than unmodified tau by LRP1-expressing cells (Fig. 4, *A* and *C*) consistent with their weaker affinity for LRP1. Notably, RAP failed to completely block tau internalization in LRP1-expressing cells confirming that LRP1-independent mechanisms exist for mediating tau internalization. Considerably less hyperphosphorylated tau was also internalized in LRP1-deficient cells (Fig. 4*B*). In summary, the data reveal that hyperphosphorylated tau produced in Sf9 insect cells binds less well to LRP1 and is less efficiently internalized by LRP1, confirming in a cell-based assay the results of the *in vitro* protein–protein interaction assay. We also investigated the internalization of the 3XKQ and 9XKQ mutant molecules in MEF cells. The results reveal reduced internalization of these molecules, consistent with their reduced affinity for LRP1 (Fig. 4*D*).

### ApoE4 blocks the internalization of tau

**Figure 5:**
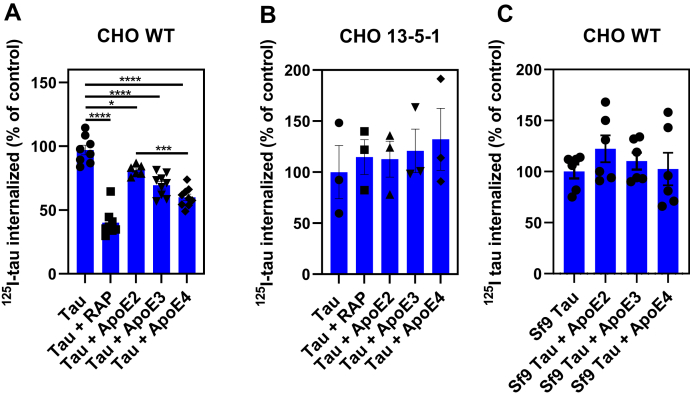
**ApoE reduces LRP1-mediated tau internalization but not hyperphosphorylated tau.** WT CHO (*A* and *C*) or LRP1-deficient CHO 13-5-1 cells (*B*) were cultured overnight in the presence of 10 μg/ml apoE2, apoE3, or apoE4. The cells were then incubated with 20 nM ^125^I-tau from *Escherichia coli* (*A* and *B*) or 100 nM of hyperphosphorylated ^125^I-tau isolated from SF9 cells (*C*) in the absence or the presence of 10 μg/ml apoE isoforms at 37 °C for 2 h. The amounts of internalized ^125^I-tau were quantified. Statistical analysis was performed using one-way ANOVA and Tukey's post hoc test (∗*p* < 0.05, ∗∗∗*p* < 0.0006, and ∗∗∗∗*p* < 0.0001). ApoE, apolipoprotein E; CHO, Chinese hamster ovary; LRP1, low-density lipoprotein receptor–related protein 1.

LRP1 is a major apoE receptor expressed in the brain, and we hypothesized that apoE may modulate LRP1-mediated tau catabolism. To test this hypothesis, we examined the effect of apoE2, apoE3, and apoE4 on the LRP1-mediated internalization of tau. In our experiments, recombinant apoE2, apoE3, or apoE4 was preincubated overnight with WT CHO cells, and following incubation, the uptake of ^125^I-labeled tau was measured. The results of this experiment reveal that only apoE3 and apoE4 isoforms significantly reduce the amount of ^125^I-labeled tau that is internalized in WT CHO cells (Fig. 5*A*). Of interest, apoE4 appears more effective in inhibiting tau internalization than the apoE2 isoform. ApoE isoforms had no effect on tau internalization in LRP1-deficent CHO 13-5-1 cells (Fig. 5*B*). We also investigated if apoE impacted the LRP1-mediated internalization of hyperphosphorylated forms of tau purified from Sf9 cells. The results (Fig. 5*C*) suggest no effect of apoE on uptake of hyperphosphorylated forms of tau, most likely because of its low affinity for LRP1 combined with the existence of additional receptors capable of internalizing hyperphosphorylated tau.

### ApoE4 binds to LRP1 with higher affinity than apoE3 or apoE2

**Figure 6:**
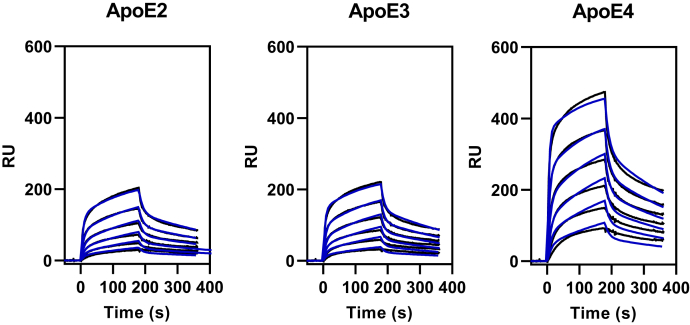
**SPR analysis of apoE isoforms binding to LRP1.** Increasing concentrations of apoE2, apoE3, or apoE4 (52, 104, 208, 417, 834, and 1668 nM) were injected over the LRP1-coupled chip. Fits of the experimental data to a bivalent binding model are shown as *blue lines*. The data shown are a representative experiment from six independent experiments that were performed. apoE, apolipoprotein E; LRP1, low-density lipoprotein receptor–related protein 1; SPR, surface plasmon resonance.

The binding of lipid-free forms of apoE isoforms to LRP1 is known to be much weaker than their binding to the very low-density lipoprotein receptor (57), but data quantifying the interaction of apoE isoforms with LRP1 have not been reported. To determine if the effects of apoE isoforms on tau catabolism correlate with their affinity for LRP1, the binding of lipid-free apoE isoforms to LRP1 quantified by SPR experiments. We fit the association and dissociation data simultaneously to a global bivalent-binding model as we have previously done for other LRP1 ligands (53, 54, 58). The results confirm that the experimental data are well described by this binding model (Fig. 6). Kinetic data from the best fit are summarized in Table 1 and reveal that the *K*_*D*_ of apoE4 for LRP1 is 202 ± 51 nM, whereas apoE3 is 456 ± 48 nM and apoE2 is 529 ± 75 nM (Table 1). Importantly, the values for the equilibrium-binding constant, *K*_*D*_, derived from kinetic analysis are close to the *K*_*D*_ value determined by equilibrium analysis of the SPR data (Table 1).

**Table 1:** Kinetic^a^ and equilibrium constants for the binding of lipid-free apoE isoforms to LRP1

Protein	k_a1_ (M^−1^ s^−1^)	k_d1_ (1/s)	k_a2_ (1/s)	k_d2_ (1/s)	b*K*_*D*_ (nM)	c*K*_*D*_ (nM)
dApoE2	3.4 ± 0.4 × 10^4^	8.6 ± 0.5 × 10^−2^	1.0 ± 0.2 × 10^−2^	2.8 ± 0.4 × 10^−3^	529 ± 75	493 ± 86
dApoE3	4.3 ± 0.8 × 10^4^	9.0 ± 0.7 × 10^−2^	9.6 ± 0.1 × 10^−3^	2.6 ± 0.2 × 10^−3^	456 ± 48	398 ± 67
dApoE4	6.2 ± 0.7 × 10^4^	6.5 ± 0.4 × 10^−2^	9.9 ± 0.3 × 10^−3^	2.7 ± 0.3 × 10^−3^	202 ± 51	236 ± 101

a Kinetic constants were obtained by fitting the data to a bivalent binding model.

b The equilibrium binding constant *K*_*A*_ was calculated using the following equation: *K*_*A*_ = (k_a1_/k_d1_) ∗ (1 + (k_a2_/k_d2_)), and *K*_*D*_ was calculated as: *K*_*D*_ = 1/*K*_*A*_.

c Calculated from equilibrium SPR measurements, in which Req was determined by fitting the association data to a pseudo first-order process to determine Req.

d Six independent experiments were performed, and the values shown are the average ± SD.

### LRP1 provides a mechanism of uptake that supports tau proteopathic seeding in the cytoplasm

**Figure 7:**
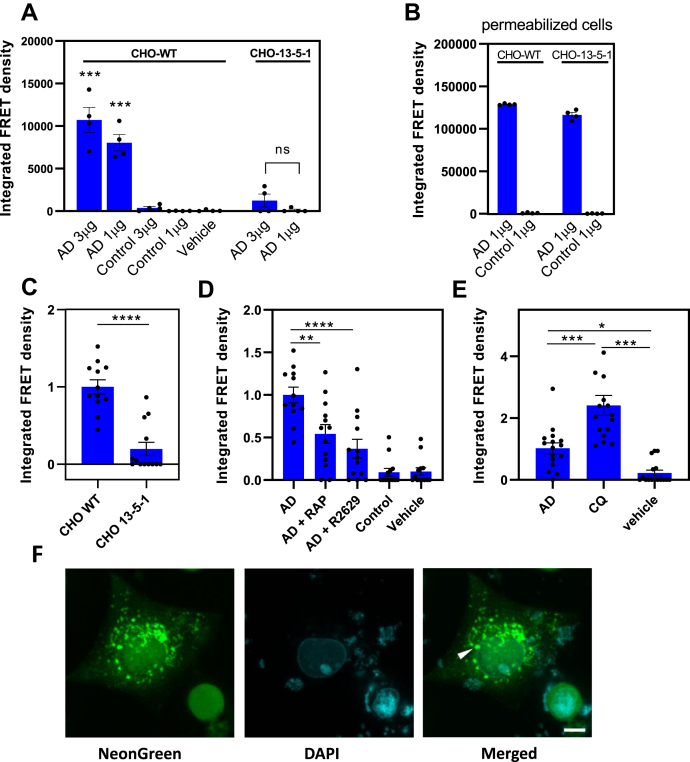
**LRP1 mediates tau seeding.***A*, CHO WT and 13-5-1 cells were transfected with pcDNA3 plasmid containing a construct that encoded residues 344 to 378 of human P301L mutant tau fused to either mTurquoise2 or mNeonGreen and then incubated with 1 to 3 μg of human brain homogenate from a patient with AD or from a healthy control for 24 h. *B*, as a positive control, 1% Lipofectamine 2000 was added to the wells. *A* and *B*, tau seeding was quantified by multiplying the percent of FRET-positive cells by the median florescence intensity of those cells. Each condition was performed in at least quadruplicate, and data were analyzed using FlowJo software. Means ± SEM, one-way ANOVA followed by Tukey's multiple comparisons test ∗∗∗*p* < 0.0001 compared with vehicle control. *C*, transfected CHO WT and 13-5-1 cells were incubated with HMW-SEC fractions from human brain of a patient with AD, shown are means ± SEM, *t* test; n = 12, ∗∗∗∗*p* < 0.0001. *D*, CHO WT cells transfected with the tau FRET reporter system were incubated with HMW-SEC fractions from human brain of a patient with AD in the presence or the absence of 1 μM RAP or anti-LRP1 IgG (R2629). *E*, CHO WT cells transfected with the tau FRET reporter system were incubated with HMW-SEC fractions from human brain of a patient with AD in the absence or the presence of 100 μM chloroquine (CQ) (means ± SEM, one-way ANOVA followed by Tukey's multiple comparisons test ∗∗∗*p* < 0.0001, ∗*p* < 0.03). *F*, representative images of foci of aggregated FRET reporter in CHO WT cells incubated with HMW-SEC tau, showing native florescence of mNeonGreen. The scale bar represents 10 μm. AD, Alzheimer's disease; CHO, Chinese hamster ovary; HMW-SEC, high–molecular weight seeding-competent; LRP1, low-density lipoprotein receptor–related protein 1; RAP, receptor-associated protein.

LRP1 efficiently mediates tau uptake and degradation of recombinant forms of the protein, and certain post-translational modifications appear to diminish tau–LRP1 interactions. Despite this, it is still possible that internalized tau can efficiently induce tau seeding resulting from escaping degradation in the endosomal/lysosomal pathways. Thus, key to understanding the potential role of LRP1s in tau propagation across cells is whether LRP1 mediates, either directly or indirectly, the uptake of human brain–derived tau from patients with AD and facilitates escape from endosomal/lysosomal systems, providing misfolded tau access to the cytoplasm to lead to seeding of endogenous tau. To determine if the LRP1-mediated uptake of pathological forms of tau results in tau seeding, we conducted experiments in which brain samples isolated from a Braak VI AD patient and a healthy control were incubated with CHO WT or CHO 13-5-1 cells that had been transfected with a tau seeding bioreporter construct. Prior studies have biochemically characterized these isolated samples and have revealed that they are readily taken up by mouse primary cortical neurons and *in vivo* to promote tau seeding (12). To detect tau seeding, we used a FRET-based biosensor assay analogous to that described by Holmes *et al.* (59) by transfecting both cell lines with a pcDNA3 plasmid containing a construct that encoded residues 344 to 378 of human P301L mutant tau fused to either mTurquoise2 or mNeonGreen. The results of this experiment reveal that incubation of CHO WT cells with brain extract from a patient with AD induces tau seeding as revealed by increased FRET, whereas incubation of brain extract from a healthy control has little effect on tau seeding (Fig. 7*A*). In contrast, incubation of LRP1-deficient CHO 13-5-1 cells with brain homogenate from patients with AD results in only marginally detectable amount of tau seeding. As a control for these experiments, when the plasma membrane of either cell line was permeabilized with lipofectamine, tau seeding occurred (Fig. 7*B*) to an equivalent extent.

CHO WT cells also demonstrate tau seeding induced by high–molecular weight (HMW) seeding-competent (SEC) tau fractions of brain extract from patients with AD, whereas tau seeding is significantly reduced, although still measurable, in LRP1-deficient CHO 13-5-1 cells (Fig. 7*C*). In WT CHO cells, the extent of tau seeding resulting from incubation with HMW-SEC tau fractions of brain extracts from patients with AD is reduced in the presence of RAP and anti-LRP1 antibodies (R2629) (Fig. 7*D*). Together, these experiments reveal that LRP1 supports the uptake and endolysosomal escape of pathological forms of tau resulting in tau seeding. We also noted that in CHO WT cells, incubation with CQ significantly increased tau seeding when the cells were incubated with HMW-SEC fractions from brain extracts of patients with AD (Fig. 7*E*). This suggests that preventing tau degradation in the lysosome enhances the likelihood that SEC tau escapes to the cytoplasm where it can interact with the biosensor molecule. Representative immunofluorescence images of seeded tau foci are shown in Figure 7*F*.

### ApoE isoforms promote tau seeding

**Figure 8:**
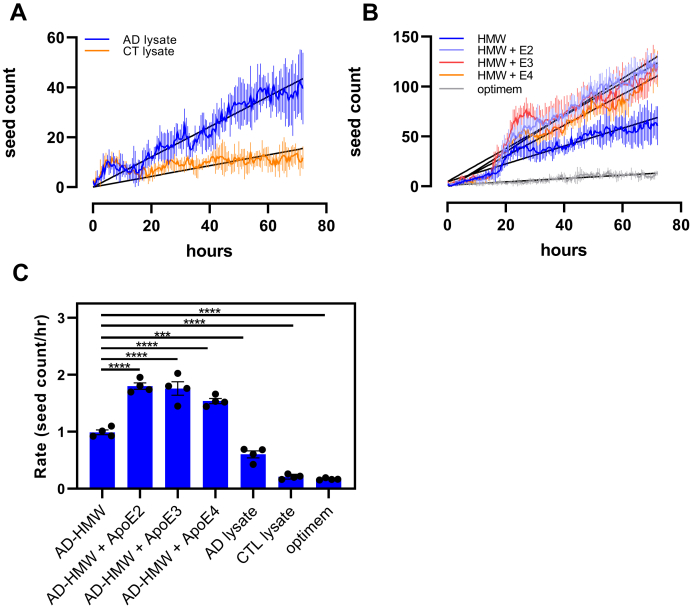
**ApoE isoforms increase the rate of seeding in stably transfected CHO WT cells**. CHO WT cells were stably transfected with pcDNA3 plasmid containing a construct that encodes residues 344 to 378 of human P301L mutant tau fused to either mTurquoise2 or mNeonGreen. *A*, the cells were incubated with lysates from patients with AD (AD) or age-matched controls (CTL), and seeding was monitored as a function of time. *B*, cells incubated with HMW-SEC fractions from human brain AD patient in the absence or the presence of apoE2, apoE3, or apoE4. Linear regression analysis was performed (*black lines*) to estimate the rate of seeding. (HMW, *r*^2^ = 0.9274; HMW + ApoE2, *r*^2^ = 0.9688; HMW + ApoE3, *r*^2^ = 0.9215; HMW + AppE4, *r*^2^ = 0/9574). *C*, rates of seeding are plotted for each treatment. Shown are means ± SEM. n = 4, one-way ANOVA followed by Tukey's multiple comparisons test to AD-HMW. ∗∗∗∗*p* < 0.0001 and ∗∗∗*p* < 0.0009. AD, Alzheimer's disease; ApoE, apolipoprotein E; CHO, Chinese hamster ovary; HMW-SEC, high–molecular weight seeding-competent.

To determine if apoE isoforms modulate tau seeding, we stably transduced CHO WT cells with a lentiviral vector containing a construct that encoded residues 344 to 378 of human P301L mutant tau fused to either mTurquoise2 or mNeonGreen. This allowed us to measure seeding as a function of time in live cells. When extracts from patients with AD were incubated with the cells, we noted a linear increase in aggregation that was greatly diminished compared with when the cells were incubated with extracts from age-matched controls (Fig. 8*A*). These cells were also incubated with HMW-SEC fractions isolated from patients with AD in the absence and presence of ApoE2, ApoE3, or ApoE4 (Fig. 8*B*). Since the time-dependent changes in aggregate formation in these cells are approximated by a linear change, we performed linear regression analysis on the data to estimate the rate of change. These values are plotted in Figure 8*C* and reveal that ApoE isoforms increase the rate of tau seeding induced by HMW-SEC fractions isolated from AD brains.

### LRP1 increases tau seeding in human embryonic kidney 293T cells

**Figure 9:**
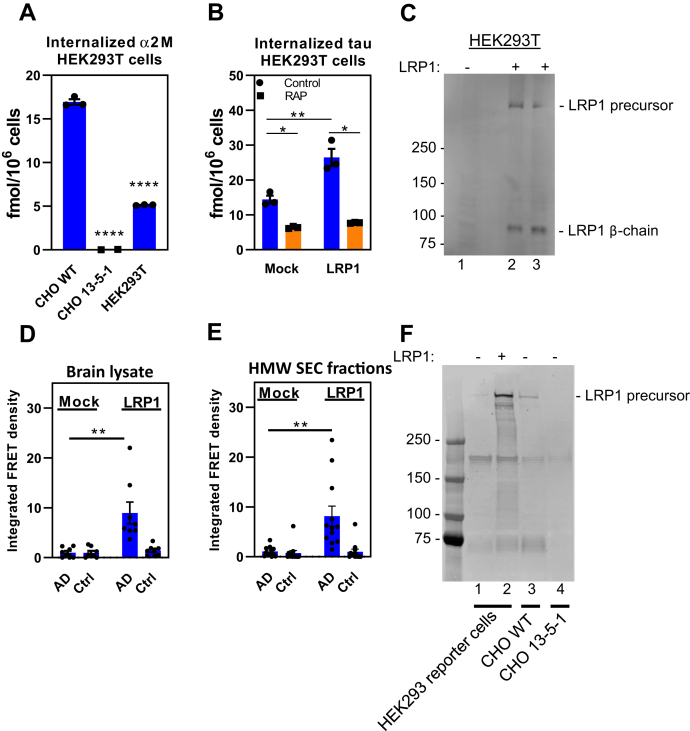
**LRP1 mediates tau seeding in HEK293T cells.***A*, CHO WT, 13-5-1, and HEK293T cells were incubated with 5 nM ^125^I-labeled a_2_M∗ for 2 h, and the amount internalized quantified. *B*, HEK293T cells were mock transfected or transfected with *LRP1* plasmid. About 24 h after transfection, cells were incubated with ^125^I-tau (20 nM) in the presence or the absence of 1 μM RAP for 2 h, and internalized tau was quantified (n = 3, technical replicates). *C*, immunoblot of cells used in (*B*) with anti-LRP1 IgG. HEK293T FRET reporter cells were transfected with *LRP1* and then incubated with (*D*) human brain homogenate from an Alzheimer's patient (n = 8, two biological replicates containing four technical replicates each) or (*E*) with HMW-SEC fractions from AD patient brain (n = 12, three biological replicates containing four technical replicates each). *F*, immunoblot of HEK283T reporter cells along with CHO WT and CHO LRP1-deficient CHO 13-5-1 cells (as controls). ((*A*) Means ± SEM, one-way ANOVA or (*B*, *D*, and *E*) two-way ANOVA followed by Tukey's multiple comparisons test. ∗*p* < 0.05, ∗∗*p* < 0.01, and ∗∗∗∗*p* < 0.0001). AD, Alzheimer's disease; CHO, Chinese hamster ovary; HEK293T, human embryonic kidney 293T; HMW-SEC, high–molecular weight seeding-competent; LRP1, low-density lipoprotein receptor–related protein 1; RAP, receptor-associated protein.

Human embryonic kidney 293T (HEK293T) cells that stably express the P301S FRET biosensor are commonly used to assay tau seeding activity. HEK293T cells express low amounts of functional LRP1 and only internalize small amounts of α_2_M∗ (Fig. 9*A*) with very little LRP1 detected by immunoblot analysis (Fig. 9*C*, *lane 1*). HEK293T cells transfected with human LRP1 internalize increased levels of tau in a process inhibited by RAP (Fig. 9*B*). In this experiment, effective transfection of LRP1 was confirmed by immunoblot analysis (Fig. 9*C*, *lanes 2* and *3*). In the final series of experiments, we utilized HEK293T biosensor cells (59) to determine if LRP1 can mediate tau seeding in these cells. These cells were generated by transfection with two tau constructs in which tau was fused to cyan fluorescent protein or yellow fluorescent protein. These biosensor cells result in FRET upon tau aggregation to form fibrils. When these cells were incubated with brain lysate from patients with AD (Fig. 9*D*) or with HMW-SEC fractions isolated from brains of patients with AD (Fig. 9*E*) in the absence of the protein transduction agent (lipofectamine) usually used in these assays, no tau seeding was detected. In contrast, expression of LRP1 in transfected HEK293T biosensor cells reveal a significant increase in tau seeding induced by brain lysates (Fig. 9*D*) or HMW-SEC fractions from AD brains (Fig. 9*E*), again in the absence of protein transduction reagents. Expression of LRP1 in these cells was confirmed by immunoblot analysis (Fig. 9*F*, *lane 2*).

## Discussion

LRP1 is an endocytic receptor that traffics ligands from the cell surface to endosomal compartments, where the ligands are typically sorted into lysosomal compartments and degraded. The overall objective of the current investigation was to quantify the extent to which tau is delivered to lysosomal compartments upon association with LRP1 and to determine if LRP1 can promote tau seeding, an event that is believed to occur only if seed competent tau escapes degradation in the lysosome and accesses the cytoplasm. We also compared the LRP1-binding properties of various forms of recombinant tau as well as post-translationally modified recombinant forms of tau. Our results demonstrate that LRP1 efficiently mediates the delivery of tau to lysosomes where the ligand is degraded. Post-translational modifications of relevance to AD-derived tau diminish binding affinity of tau to LRP1 and diminish uptake. However, in contrast to other LRP1 ligands, we also demonstrate that LRP1 mediates leakage of SEC tau species derived from human Alzheimer brain samples into the cytoplasm where it can support seeding in a cell-based bioactivity assay.

Using cell lines deficient in LRP1 or antagonists to block LRP1 function, we quantified the uptake and cellular processing of ^125^I-labeled tau in multiple cell lines. Our results reveal that while cells deficient in LRP1 are not as effective in internalizing tau, residual tau internalization does occur suggesting the existence of LRP1-independent pathways for tau internalization. Our results support the recent published studies demonstrating that LRP1 can mediate the internalization of tau using flow cytometry approaches (21) and studies using whole genome CRISPR screens that identified LRP1 as a top hit mediating extracellular tau uptake by human neurons (60). Furthermore, in excellent agreement with the data of Rauch *et al.* (21), our data confirm that the MBD of tau binds tightly to LRP1. In addition, our data reveal that ligand-binding clusters II, III, and IV of LRP1 are all capable of binding tau, which differs from the results of Rauch *et al.* who used LRP1 minireceptors expressing various LRP1 clusters. This discrepancy may be due to the different systems that used a protein–protein interaction assay *versus* a cell-based uptake system, combined with previous observations that LRP1 minireceptors, especially those expressing clusters II and III, are not effectively delivered to the cell surface (56). It is somewhat unusual for an LRP1 ligand to be recognized by all three LRP1 ligand-binding repeats and suggests that tau may bind to dimeric forms of LRP1 on cells, or to more than one cluster at a time, which would result in increased affinity *via* avidity effects.

We observe that monomeric 4R tau binds to LRP1 with higher affinity than 3R tau, and that the isolated MBD has a strong affinity for LRP1 as well, consistent with the idea that this portion of the tau molecule represents the primary binding domain (Fig. 3). Interestingly, this domain is also the heart of the fibrillar species found in both NFTs (which contain both 3R and 4R tau) as well in other tauopathies, where the 4R isoform predominates. These results suggest that LRP1-mediated binding and uptake may play a role in multiple tauopathies as well as in AD, although of course the conformation of tau in these various disorders is sufficiently different that direct comparisons may be necessary.

Interestingly, the study by Rauch *et al.* (21) employed a unique adeno-associated virus construct (expressing GFP-2A-hTau) that is capable of discriminating between transduced cells and cells internalizing secreted tau (11) to demonstrate that neuronal LRP1 expressed in the brain of mice readily internalizes neuronally secreted tau. This assay does not distinguish among the various post-translationally–modified forms of tau that are present in the central nervous system and does not measure tau aggregation in the recipient neuron, that is, the seeding of endogenous tau. To determine if LRP1 can promote tau seeding, we incubated LRP1-expressing and LRP1-deficient cells with human brain homogenates from AD and healthy controls and used a FRET-based biosensor assay to detect seeding. These studies revealed that LRP1-expressing cells promoted tau seeding when incubated with homogenates from patients with AD but not homogenates from healthy controls. In addition, expression of LRP1 in HEK293T cells, which have low levels of LRP1, results in tau seeding when incubated with human brain homogenates from patients with AD. Together, these results confirm that LRP1 expression promotes tau seeding.

These results highlight a paradoxical situation whereby on one hand LRP1 mediates the internalization of monomeric forms of tau, which leads to degradation of tau in lysosomal compartments. The binding and uptake properties of tau with post-translational modifications corresponding to those seen to accumulate in AD, multiple phosphorylations, and acetylations, appear to diminish LRP1-assocaited binding and uptake. On the other hand, LRP1 appears to nonetheless participate in the internalization of pathogenic post-translationally–modified forms of tau, which can then induce cytoplasmic seeding of tau. We hypothesize that LRP1-mediated uptake of monomeric tau represents a normal physiological pathway by which physiologically secreted tau is catabolized. We also suggest that LRP1 participates, either directly or indirectly, in mediating the uptake of pathological forms of tau that lead to tau seeding, which would require endolysosomal escape of tau. As a precedent for this, the LRP1 ligand, *Pseudomonas* exotoxin A, is cleaved within endosomal compartments releasing a 37 kDa domain that is translocated to the cytosol where it inhibits ADP ribosylation of elongation factor 2 (61). Interestingly, altering the endolysosomal pH with CQ increases the escape of tau seeds to the cytosol, possibly by extending the residence time in a susceptible exit compartment. It remains unknown whether coreceptors exist that facilitate the uptake and endolysosomal escape of multimeric or other post-translationally–modified forms of tau.

Our data highlight that the interaction of tau and LRP1 may be influenced by post-translational modifications in tau since phosphorylated forms of tau bound much more weakly to LRP1 than unphosphorylated tau forms. The weaker affinity of these pathologic forms of tau for LRP1 may have important consequences on trafficking of tau by allowing modified tau to dissociate from LRP1 within early endosomes to facilitate endosomal escape. It is interesting to highlight in this regard that experiments employing hybrid constructs of *Pseudomonas* exotoxin A noted an inverse correlation between LRP1-binding affinity and toxicity (62), which requires endosomal escape of the toxin. Thus, a hybrid toxin containing a domain with high affinity for LRP1 was much less toxic to cells, likely because of more effective delivery to lysosomal compartments for degradation. In contrast, hybrid toxins with a weaker affinity for LRP1 were much more toxic revealing increased capacity for endosomal escape.

**Figure 10:**
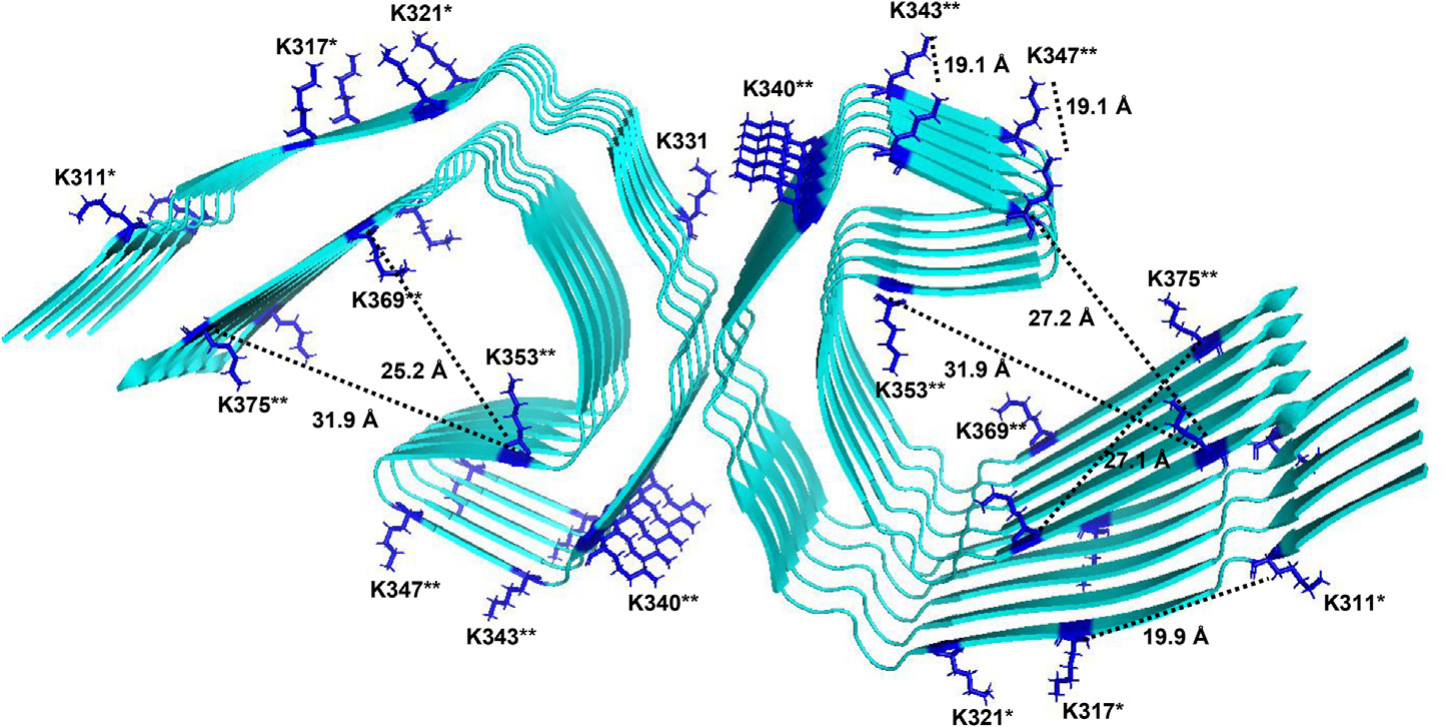
**Surface-accessible lysine residues available for LRP1 binding on tau protofilament from Alzheimer's disease.***Ribbon* diagram of tau filament core (Protein Data Bank: 5O3L) (70) showing accessible surface areas (ASAs) for lysine residues available for interacting with LRP1 (ASA > 0.5). ASA was calculated from the coordinates in Protein Data Bank 5O3L using Volume, Area, Dihedral Angle Reporter, version 1.8 (71). Distances between the a-carbon of lysine residues were determined using PyMOL software. ∗Lysine resides mutated in 3XKQ tau mutant; ∗∗additional lysine residues mutated in 9XKQ mutant. LRP1, low-density lipoprotein receptor–related protein 1.

Early studies recognized the importance of basic amino acids on the ligand that seemed critical for LRP1 receptor binding (52, 63, 64, 65, 66). When the structure of two complement-like repeats (CRs) from the LDL receptor in complex with the third domain of RAP (67) was solved, a canonical model for ligand binding to LRP1 was suggested in which acidic residues from each CR formed an acidic pocket for in which two lysine side chains (K256 and K270) from RAP are docked. The acidic pocket on the receptors is stabilized by a calcium ion, and the interaction with ligand is strengthened by an aromatic residue on the receptor that forms van der Waals interactions with the aliphatic portion of the lysine residue that is docked in the “acidic pocket” (67) and by other lysine residues on ligands that form weak electrostatic interactions (68). Interestingly, Rauch *et al.* (21) found that chemical modification of lysine residues on tau prevented uptake of tau. An evaluation of the cryo-EM structure of paired helical filaments of tau isolated from AD brains (69) revealed six surface-accessible lysine residues (70) per monomer, which are displayed in Figure 10. Optimal distances between lysine residues for docking into CR repeats can be estimated from known structures of various CR repeats from this family of receptors, with a range of ~14 to 41 Å and an average of 26 Å (Table 2). Several surface-accessible lysine residues on the filament core of tau isolated from patients with AD are within this optimal distance for LRP1 binding, which supports the observation that LRP1 is capable of enhancing tau seeding. We mutated several of these lysines to glutamines, which mimics tau acetylation, and found markedly reduced tau binding to LRP1.

**Table 2:** Distances between CRs on LDL receptor family members^a^

Protein Data Bank number	Receptor	Repeats	Distance, Å
1f5y	LDLR	CR1, 2	27.6
2fy1	LRP1	CF5, 6	21.5
2lgp	LDLR	CR4, 5	31.8
6byv	VLDLR	CR2, 3, 4	27.1; 24.1
2fcw	LDLR/RAP D3	CR3, 4	21.2
1n7d	LDLR ectodomain, endosomal pH	CR2, 3, 4, 5, 6, 7	31.7; 20.3; 26.4; 41.1; 14.1

a Distances between Ca^2+^ ions that stabilized the CR repeats.

We also found that all isoforms of apoE inhibited the uptake of physiological forms of tau, but not hyperphosphoryated forms of tau, in CHO WT cells with apoE4 having the greatest impact, consistent with its higher affinity for LRP1. Using a flow cytometry–based internalization assay, Rauch *et al.* (21) also observed that apoE reduced tau internalization but found no differences across isoforms. The difference between their results and those of the current study may result from assay conditions. While the effect of apoE could be due to direct competition of binding to LRP1 with tau, it could also have indirect effects as it has been observed that apoE4 traps LRP1 within intracellular compartments reducing surface levels (71) and selectively impairs the recycling and surface expression apoER2, another LDL receptor family member (72). Interestingly, ApoE4 is known to exacerbate tau-mediated neurodegeneration in P301S tau transgenic mice on a human ApoE4 background (73). Further, a patient was recently identified who inherited an autosomal-dominant E280A mutation in presenilin 1 that remained disease free because of homozygosity of APOE3ch gene variant (74). The study concluded that the APOE3ch homozygote conferred resistance to the clinical onset of AD by limiting tau pathology even in the face of high amyloid-β plaque burden (74). This mutant form of ApoE is impaired in its ability to bind to LDL receptor family members (75) and thus implies a connection between apoE, LRP1 (or other LDL receptor family members), and tau catabolism that might give insight into this interesting observation.

It is striking that we found that apoE isoforms enhance tau seeding when reporter cells were incubated with HMW-SEC fractions from patients with AD. These data suggest that mechanisms associated with the uptake of pathological forms of tau differ considerably from those associated with the uptake of physiological forms of tau. A recent study by Dujardin *et al.* (76) argues that molecular diversity of tau contributes to AD heterogeneity and that some post-translational modification sites are associated with both enhanced seeding activity and worse clinical outcomes, whereas others are not. Understanding the impact of various post-translational modifications on LRP1-mediated tau processing may be key to understanding the clinical and histopathological diversity of AD. We find it a notable observation that hyperphosphorylated forms of tau, which are considered pathogenic, bind less efficiently to LRP1 and are internalized less efficiently by LRP1 and that apoE4, an isoform that is linked to early onset AD, is more effective in reducing the capacity of LRP1 to internalize tau.

In summary, our data suggest that LRP1-mediated endocytosis of tau represents a physiological pathway that may be critical for normal neuronal function. On the other hand, our data also suggest that LRP1 participates in the uptake of pathological forms of tau leading to tau seeding. Since LRP1 mediates both tau degradation and tau seeding, the role of LRP1 in tau pathology is nuanced and likely varies depending on the cellular environment and the species of tau involved. Differentiation of these two pathways will be critical to understand the roles of this receptor in AD pathology. LRP1 is a central receptor that regulates trafficking and metabolism of several important molecules linked to AD, which include APP (22, 23, 24) and β-amyloid (27, 28), tau, and apoE (77, 78). Our work positions LRP1 as an unprecedented molecular point of convergence for the pathological hallmarks of AD. Understanding the individual pathways of each molecule and how they interconnect to LRP1 is key to the development of potential therapeutic intervention in AD and potentially other tauopathies.

## Experimental procedures

### Cells

Human primary fibroblasts (WI-38) and HEK293T cells were purchased from American Type Culture Collection (ATCC) and maintained in Dulbecco's modified Eagle' medium (DMEM; Corning 10-013-CV) supplemented with 10% fetal bovine serum (FBS; Sigma F-4135). CHO K1 (WT CHO) and CHO 13-5-1 cells (34) were maintained in DMEM/Ham's F12 with l-glutamine (DMEM/F12; Corning 10-090-CM) supplemented with 10% FBS. CHO cells deficient in xylosyltransferase (CHO-745) (38) provided by Jeffrey Esko were maintained in Kaighn's modification of Ham's F-12 medium (ATCC 30-2004) supplemented with 10% FBS. SH-SY5Y neuroblastoma cells were maintained in DMEM/F12 supplemented with 10% FBS. MEF and PEA-13 cells were maintained in DMEM/F12 supplemented with 10% FBS. The Tau RD P301S FRET Biosensor embryonic kidney 293T cells (ATCC CRL-3275) provided by Marc Diamond were maintained in DMEM supplemented with 10% FBS. All cells were cultured with 1× penicillin–streptomycin (Corning 30-002-CI) and maintained at 37 °C and 5% CO_2_ in a humidified atmosphere.

### Proteins, antibodies, and plasmids

Full-length human LRP1 was purified from placenta (79). RAP was expressed in *E. coli* (80). The mouse anti-LRP1 monoclonal antibody 5A6 was used to recognize the 85 kDa light chain of LRP1, and the rabbit anti-LRP1 polyclonal (R2629) antibody was used to inhibit ligand binding to LRP1 as previously described (81). Full-length tau (2N4R; SP-495) and tau MBD (SP-496) were purchased from R&D Systems. Recombinant human LRP1 cluster II, III, and IV Fc chimera proteins were produced by Molecular Innovations. Recombinant human apoE2, apoE3, and apoE4 expressed in *E. coli* were purchased from either MBL International Corporation (JM-4760, JM-4696, and JM-4699) or Peprotech (350-12, 350-02, and 350-04). His-tagged recombinant human tau variants 2N4R, 2N3R, and mutated proteins were expressed in *E. coli* and purified. His-tagged phosphorylated tau was produced in SF9 cells. Cells were not treated with phosphatase inhibitor during production, resulting in an intermediate tau phosphorylation state (50). 10× 175-cm^2^ SF9 cells with 80 to 90% confluency were infected with P3 or P4 recombinant baculovirus (MOI 5-10) and incubated at 27 °C for 48 to 72 h. Cells were pelleted at 500*g* for 5 min and resuspended in 30 ml lysis buffer containing 50 mM Tris–HCl, 100 mM NaCl, 10% glycerol, 5 mM imidazole, 0.5 mM tris(2-carboxyethyl)phosphate, 0.1 mM PMSF, benzonase 30 U/ml, and 1× Halt protease, and phosphatase inhibitor cocktail (Thermo Scientific 1861282). Cells were crushed in a French press twice, suspension was boiled for 20 min in a 100 °C water bath, cooled down on ice for 15 min, and centrifuged at 15,000*g* for 30 min to remove debris. Lysate was run on a HisTrap affinity column, and collected fractions were dialyzed into PBS containing 0.5 mM tris(2-carboxyethyl)phosphate. His tag was removed by enzymatic cleavage. 2N4R tau harboring the 6A or 6E mutations was generated by converting T181, S199, S202, S396, S400, and S404 to alanine (6A mutant) or glutamic acid (6E mutant). 3XKQ and 9XKQ 2N4R tau were generated in *E. coli* by mutating lysine residues 311, 317, and 321 or lysine residues 311, 217, 321, 340, 343, 347, 353, 369, and 375 to glutamine residues. Alpha-2-macroglobulin was purchased from Athens Research and was activated by methylamine as described (79). Full-length human *LRP1* was cloned into pN1 expression vector by VectorBuilder.

### Tau internalization and degradation assay

Cellular internalization assays were conducted as previously described (22, 23, 34). Twelve-well culture dishes were seeded with WI-38 (0.5 × 10^4^ cells per well), CHO (2 × 10^5^ cells per well), or HEK293T (9.5 × 10^4^ cells per well) cells. Cells were cultured overnight in DMEM (WI-38) or DMEM/F12 (CHO) with 10% FBS and 1× penicillin–streptomycin. The following day, cells were incubated in assay media (DMEM supplemented with 1.5% bovine serum albumin and 20 mM Hepes) for 1 h and then incubated with assay media containing 20 nM ^125^I-labeled tau (2N4R; R&D Systems, Inc; SP-495) in the presence or absence of 1 μM RAP for specified times. In some experiments, ^125^I-labeled tau was coincubated with 100 μM CQ (Sigma C6628), 20 μg/ml heparin (Sigma H-3125), or 300 μg/ml R2629. For experiments assessing a single cycle of tau uptake, MEF or PEA-13 cells were plated at 2 × 10^5^ cells/well and incubated overnight as described previously. Cells were then incubated for 2 h at 4 °C in assay media containing 20 nM tau with or without 1 μM RAP. After incubation, cells were washed with Dulbecco's PBS, and fresh assay media maintained at 37 °C was added to the cells to trigger endocytosis. Cells were incubated at 37 °C for designated times, and then collected assay cell–associated, internalized, and degraded tau. RAP-sensitive tau uptake was calculated by subtracting the RAP-inhibitable uptake from the total.

### Transfections

HEK293T cells were plated at 9.5 × 10^4^ cells per well in a 12-well culture dish in culture media without antibiotics. About 24 h after plating, cells were transfected with *LRP1* in pN1 expression vector using 0.75 μg DNA per well *via* PEI transfection reagent at a ratio of 6 μl PEI:1 μg DNA. Transfection with empty vector was used as control. Cells were incubated overnight in transfection reagent, and 24 h after transfection, the tau internalization assay was performed as described previously.

### SPR

Binding of tau isoforms 2N4R, 2N3R, and 2N4R tau harboring the 6A and 6E mutations, hyperphosphorylated 2N4R tau produced in Sf9 cells, 3XKQ and 9XKQ 2N4R tau, and apoE to LRP1 were assessed using a Biacore 3000 optical biosensor system (GE Healthcare Life Sciences) essentially as described (54, 58). Single kinetic titrations were performed by serial injections from low to high concentration (3.8, 11.5, 34.4, 103.3, and 310 nM) with a 3.5-min injection time. Between sample runs, sensor chip surfaces were regenerated with 15-s injections of 0.5% SDS at a flow rate of 100 μl/min. In some experiments, sensor chip surfaces were regenerated with a low pH solution of 100 mM phosphoric acid (pH ~2.5).

### Kinetic analysis of SPR data

Kinetic data were analyzed to a bivalent model (presented below) using BIAevaluation software (Biacore AB) as previously described (54):

**Figure:**
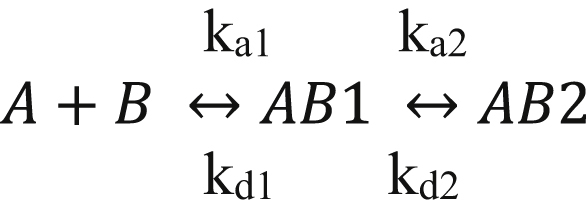

Where A represents apoE isoforms, B represents LRP1, and AB1 represents apoE:LRP1 complexes in which a single region of the ligand is complexed with LRP1, whereas AB2 represents the bivalent apoE:LRP1 complex. To facilitate the fitting process, estimates for k_d1_ and k_d2_ were obtained by fitting the dissociation data globally to a two-exponential decay model. These values were then used as initial estimates in the fitting process.

### Tau seeding FRET biosensor assay

Human brain homogenates were prepared from an AD Braak VI brain and one healthy control brain from the Massachusetts Alzheimer's Disease Research Center Brain Bank. Briefly, 100 mg of frontal cortex tissue (Brodmann area 8/9) were thawed and homogenized in 500 μl of PBS with protease inhibitor (Roche) by 30 up and down strokes in a glass Dounce homogenizer. The homogenate was centrifuged at 10,000*g* for 10 min at 4 °C. The supernatant was aliquoted, and a bicinchoninic acid assay (Thermo Scientific Pierce) was performed according to manufacturer's instructions to quantify total protein concentration. Soluble HMW-SEC tau was isolated from homogenate using size exclusion chromatography on a Superdex200 10/300GL column (#17-5175-01; GE Healthcare) as described previously (12). Total tau concentration was measured by ELISA (#K15121D; Meso Scale Discovery). The seeding assay that had been previously described was adapted for the present study (59, 82). CHO WT and CHO 13-5-1 cells were reverse transfected with a pcDNA3 plasmid containing a construct that encoded the 344 to 378 residues of human P301L mutant tau fused to mTurquoise2, a self-cleaving 2A peptide, and 344 to 378 of human P301L mutant tau fused to mNeonGreen in Costar Black (Corning) clear bottom 96-well plates, using trans-IT X2 reagent (Mirus) according to manufacturer's protocol. Cells were seeded at 60,000 cells/well and transduced with 100 ng DNA/well. The next day, transfection media were replaced with 50 μl of Opti-MEM containing 50 ng of total tau from human brain extract in the presence or absence of RAP. For positive controls, brain extracts were incubated with 1% lipofectamine 2000 for 15 min and then added to the wells, forcing the entry of tau seeds. Each condition was tested at least in quadruplicate. Cells were incubated with lysates for 24 to 28 h. Cells were then collected using trypsin and transferred into 96-well U-bottom plates (Corning) using 10% FBS culture media to neutralize trypsin. Cells were pelleted at 1200*g* for 10 min, resuspended in cold 2% paraformaldehyde for 10 min, pelleted at 1200*g*, and resuspended in 200 μl of PBS. Samples were run on the MACSQuant VYB (Miltenyi) flow cytometer for the quantification of turquoise fluorescence and FRET. Tau seeding was quantified by multiplying the percent of FRET-positive cells by the median fluorescence intensity of those cells, as described previously (9). About 40,000 cells per well were analyzed. Data were analyzed using FlowJo software (BD Biosciences). For experiments in the Tau RD P301S FRET Biosensor embryonic kidney 293T cells, cells were plated at 30,000 cells/well in 1/20 poly-d-lysine (Merck Millipore) precoated Costar Black (Corning) clear bottom 96-well plates. After 24 h, cells were transfected with 100 ng/well of LRP1 in pN1 expression vector with 0.4 μl/well Lipofectamine 2000 (Thermo Fisher Scientific). The following steps were similar to the assay in CHO cells. All competing reagents and drugs including RAP (1 μM), R2629 anti-LRP1 (300 μg/ml), and CQ (100 μM; Sigma–Aldrich) were incubated on cells with brain homogenate. For apoE experiments, stable clonal CHO cells expressing tau mTurquoise2–mNeonGreen FRET reporter were exposed to HMW-SEC tau (150 ng/well measured by Western blot). Cells were preincubated with recombinant apoE (10 μg/ml) for 1 h before and coincubated with recombinant apoE and HMW-SEC tau during the entire experiment.

### Live cell imaging

A stable clonal CHO line expressing tau mTurquoise2–mNeonGreen FRET reporter was plated at 8000 cell/well in a 96-well plate. After 24 h, cells were incubated with tau seeds in Opti-MEM and imaged on the GFP channel every 30 min for 72 h on a Cytation 5 live cell imager (BioTek). Images were processed on Gen5 software (BioTek) using thresholding to quantify intracellular aggregates.

### Immunofluorescence

SH-SY5Y cells were grown on eight-chamber microscope slides in DMEM/F12 supplemented with 10% FBS until subconfluent. Cells were serum starved by incubating in DMEM/F12 in the absence of FBS for 1 h prior to the experiment. 5A6 antibody and 2N4R tau were labeled using Alexa Flour 488 and 594 antibody labeling kits according to manufacturer's instructions (Life Technologies). LRP1 molecules undergoing endocytosis were labeled as previously described (32) by incubating cells with 60 nM 5A6 anti-LRP1 antibody conjugated to Alexa Flour 488 in DMEM/F12 at 37 °C for 2 h. Cells were washed to remove unbound antibody and then incubated with 40 nM tau conjugated to Alexa Flour 594 in DMEM/F12 at 37 °C for 2 h. After washing to remove unbound tau, cells were fixed in 4% paraformaldehyde for 10 min at room temperature. Slides were washed with Dulbecco's PBS, and coverslips were mounted using VectaSheild Antifade with 4′,6-diamidino-2-phenylindole (Vector laboratories H-1200). Fluorescent images were acquired using a CSU-W1 spinning disk confocal system (Nikon) in the Center for Innovative Biomedical Resources Confocal Microscopy Facility at the University of Maryland School of Medicine. Images were acquired with a 60× 1.49 numerical aperture oil-immersion objective as z-stacks with a step size of 0.1 μm and represented as maximal intensity projections along the *z*-axis in ImageJ2 software (83).

### SDS-PAGE and Western blot

Cell cultures were collected in radioimmunoprecipitation assay lysis buffer and analyzed by Western blotting as previously described. Equal amounts of protein from each sample were mixed with loading buffer with or without 100 mM/l dithiothreitol, boiled for 5 min, resolved by electrophoresis on a Novex 4 to 12% Tris-Glycine Mini Protein Gel, and transferred to polyvinylidene difluoride membranes for Western blot analysis. Membranes were blocked with Odyssey blocking buffer and incubated with anti-LRP1 (R2629 or 5A6) at a concentration of 1:1000 overnight at 4 °C. The membrane was washed three times with 0.05% Tween-20 in Tris-buffered saline, and the antibody binding to membrane was detected with IRDye 680RD or 800 antimouse or anti-rabbit IgG secondary antibody (LI-COR Biosciences) at a concentration of 1:10,000. The membrane was then washed three times with 0.05% Tween-20 in Tris-buffered saline and imaged using a LI-COR Odyssey Infrared Imaging System.

### Experimental design and statistical analysis

All results are represented as mean ± SEM or SD, as indicated. Data were analyzed for significance using two-tailed Student's *t* test, one-way ANOVA, or two-way ANOVA, with Tukey's or Sidak multiple comparisons post-tests, as indicated. A *p* value of <0.05 was set as the threshold for significance.

## Data availability

All data are contained within the article.

## Conflict of interest

The authors declare that they have no conflicts of interest with the contents of this article.

## References

[1] Hyman B.T., Van Hoesen G.W., Damasio A.R., Clifford L. (1984). Alzheimer’s disease: Cell-specific pathology isolates the hippocampal formation. Science.

[2] Serrano-Pozo A., Qian J., Monsell S.E., Frosch M.P., Betensky R.A., Hyman B.T. (2013). Examination of the clinicopathologic continuum of Alzheimer disease in the autopsy cohort of the National Alzheimer Coordinating Center. J. Neuropathol. Exp. Neurol..

[3] Braak H., Braak E. (1991). Neuropathological stageing of Alzheimer-related changes. Acta Neuropathol..

[4] Polydoro M., de Calignon A., Suárez-Calvet M., Sanchez L., Kay K.R., Nicholls S.B., Roe A.D., Pitstick R., Carlson G.A., Gómez-Isla T., Spires-Jones T.L., Hyman B.T. (2013). Reversal of neurofibrillary tangles and tau-associated phenotype in the rTgTauEC model of early Alzheimer’s disease. J. Neurosci..

[5] De Calignon A., Polydoro M., Suárez-Calvet M., William C., Adamowicz D.H., Kopeikina K.J., Pitstick R., Sahara N., Ashe K.H., Carlson G.A., Spires-Jones T.L., Hyman B.T. (2012). Propagation of tau pathology in a model of early Alzheimer’s disease. Neuron.

[6] Liu L., Drouet V., Wu J.W., Witter M.P., Small S.A., Clelland C., Duff K. (2012). Trans-synaptic spread of tau pathology *in vivo*. PLoS One.

[7] Harris J.A., Koyama A., Maeda S., Ho K., Devidze N., Dubal D.B., Yu G.Q., Masliah E., Mucke L. (2012). Human P301L-mutant tau expression in mouse entorhinal-hippocampal network causes tau aggregation and presynaptic pathology but no cognitive deficits. PLoS One.

[8] Kaufman S.K., Del Tredici K., Thomas T.L., Braak H., Diamond M.I. (2018). Tau seeding activity begins in the transentorhinal/entorhinal regions and anticipates phospho-tau pathology in Alzheimer’s disease and PART. Acta Neuropathol..

[9] DeVos S.L., Corjuc B.T., Oakley D.H., Nobuhara C.K., Bannon R.N., Chase A., Commins C., Gonzalez J.A., Dooley P.M., Frosch M.P., Hyman B.T. (2018). Synaptic tau seeding precedes tau pathology in human Alzheimer’s disease brain. Front. Neurosci..

[10] Medina M., Avila J. (2014). The role of extracellular tau in the spreading of neurofibrillary pathology. Front. Cell. Neurosci..

[11] Wegmann S., Bennett R.E., Delorme L., Robbins A.B., Hu M., McKenzie D., Kirk M.J., Schiantarelli J., Tunio N., Amaral A.C., Fan Z., Nicholls S., Hudry E., Hyman B.T. (2019). Experimental evidence for the age dependence of tau protein spread in the brain. Sci. Adv..

[12] Takeda S., Wegmann S., Cho H., Devos S.L., Commins C., Roe A.D., Nicholls S.B., Carlson G.A., Pitstick R., Nobuhara C.K., Costantino I., Frosch M.P., Muller D.J., Irimia D., Hyman B.T. (2015). Neuronal uptake and propagation of a rare phosphorylated high-molecular-weight tau derived from Alzheimer’s disease brain. Nat. Commun..

[13] Yamada K., Cirrito J.R., Stewart F.R., Jiang H., Finn M.B., Holmes B.B., Binder L.I., Mandelkow E.M., Diamond M.I., Lee V.M.Y., Holtzman D.M. (2011). *In vivo* microdialysis reveals age-dependent decrease of brain interstitial fluid tau levels in P301S human tau transgenic mice. J. Neurosci..

[14] Chai X., Dage J.L., Citron M. (2012). Constitutive secretion of tau protein by an unconventional mechanism. Neurobiol. Dis..

[15] Merezhko M., Brunello C.A., Yan X., Vihinen H., Jokitalo E., Uronen R.L., Huttunen H.J. (2018). Secretion of tau via an unconventional non-vesicular mechanism. Cell Rep..

[16] Pooler A.M., Phillips E.C., Lau D.H.W., Noble W., Hanger D.P. (2013). Physiological release of endogenous tau is stimulated by neuronal activity. EMBO Rep..

[17] Huijbers X.W., Schultz A.P., Papp K.V., Lapoint M.R., Hanseeuw X., Chhatwal X.J.P., Hedden T., Johnson X.A., Sperling X.R.A. (2019). Tau accumulation in clinically normal older adults is associated with hippocampal hyperactivity. J. Neurosci..

[18] Harrison T.M., La Joie R., Maass A., Baker S.L., Swinnerton K., Fenton L., Mellinger T.J., Edwards L., Pham J., Miller B.L., Rabinovici G.D., Jagust W.J. (2019). Longitudinal tau accumulation and atrophy in aging and Alzheimer disease. Ann. Neurol..

[19] Frost B., Jacks R.L., Diamond M.I. (2009). Propagation of tau misfolding from the outside to the inside of a cell. J. Biol. Chem..

[20] Swanson E., Breckenridge L., McMahon L., Som S., McConnell I., Bloom G.S. (2017). Extracellular tau oligomers induce invasion of endogenous tau into the somatodendritic compartment and axonal transport dysfunction. J. Alzheimers Dis..

[21] Rauch J.N., Luna G., Guzman E., Audouard M., Challis C., Sibih Y.E., Leshuk C., Hernandez I., Wegmann S., Hyman B.T., Gradinaru V., Kampmann M., Kosik K.S. (2020). LRP1 is a master regulator of tau uptake and spread. Nature.

[22] Kounnas M.Z., Moir R.D., Rebeck G.W., Bush A.I., Argraves W.S., Tanzi R.E., Hyman B.T., Strickland D.K. (1995). LDL receptor-related protein, a multifunctional ApoE receptor, binds secreted β-amyloid precursor protein and mediates its degradation. Cell.

[23] Ulery P.G., Beers J., Mikhailenko I., Tanzi R.E., Rebeck G.W., Hyman B.T., Strickland D.K. (2000). Modulation of β-amyloid precursor protein processing by the low density lipoprotein receptor-related protein (LRP). Evidence that LRP contributes to the pathogenesis of Alzheimer’s disease. J. Biol. Chem..

[24] Waldron E., Heilig C., Schweitzer A., Nadella N., Jaeger S., Martin A.M., Weggen S., Brix K., Pietrzik C.U. (2008). LRP1 modulates APP trafficking along early compartments of the secretory pathway. Neurobiol. Dis..

[25] Pietrzik C.U., Busse T., Merriam D.E., Weggen S., Koo E.H. (2002). The cytoplasmic domain of the LDL receptor-related protein regulates multiple steps in APP processing. EMBO J..

[26] Van Gool B., Storck S.E., Reekmans S.M., Lechat B., Gordts P.L.S.M., Pradier L., Pietrzik C.U., Roebroek A.J.M. (2019). LRP1 has a predominant role in production over clearance of Aβ in a mouse model of Alzheimer’s disease. Mol. Neurobiol..

[27] Shibata M., Yamada S., Ram Kumar S., Calero M., Bading J., Frangione B., Holtzman D.M., Miller C.A., Strickland D.K., Ghiso J., Zlokovic B.V. (2000). Clearance of Alzheimer’s amyloid-β1-40 peptide from brain by LDL receptor-related protein-1 at the blood-brain barrier. J. Clin. Invest..

[28] Storck S.E., Bayer T.A., Pietrzik C.U., Storck S.E., Meister S., Nahrath J., Meißner J.N., Schubert N., Di Spiezio A., Baches S., Vandenbroucke R.E., Bouter Y., Prikulis I., Korth C., Weggen S. (2016). Endothelial LRP1 transports amyloid- β 1 – 42 across the blood-brain barrier. J. Clin. Invest..

[29] Sagare A., Deane R., Bell R.D., Johnson B., Hamm K., Pendu R., Marky A., Lenting P.J., Wu Z., Zarcone T., Goate A., Mayo K., Perlmutter D., Coma M., Zhong Z. (2007). Clearance of amyloid-β by circulating lipoprotein receptors. Nat. Med..

[30] Corder E.H., Saunders A.M., Strittmatter W.J., Schmechel D.E., Gaskell P.C., Small G.W., Roses A.D., Haines J.L., Pericak-Vance M.A. (2008). Gene dose of apolipoprotein E type 4 allele and the risk of Alzheimer’s disease in late onset families. Science.

[31] Strittmatter W.J., Saunders A.M., Schmechel D., Pericak-Vance M., Enghild J., Salvesen G.S., Roses A.D. (1993). Apolipoprotein E: High-avidity binding to β-amyloid and increased frequency of type 4 allele in late-onset familial Alzheimer disease. Proc. Natl. Acad. Sci. U. S. A..

[32] Muratoglu S.C., Mikhailenko I., Newton C., Migliorini M., Strickland D.K. (2010). Low density lipoprotein receptor-related protein 1 (LRP1) forms a signaling complex with platelet-derived growth factor receptor-beta in endosomes and regulates activation of the MAPK pathway. J. Biol. Chem..

[33] Herz J., Goldstein J.L., Strickland D.K., Ho Y.K., Brown M.S. (1991). 39-kDa protein modulates binding of ligands to low density lipoprotein receptor-related protein/α2-macroglobulin receptor. J. Biol. Chem..

[34] FitzGerald D.J., Fryling C.M., Zdanovsky A., Saelinger C.B., Kounnas M., Winkles J.A., Strickland D., Leppla S. (1995). Pseudomonas exotoxin-mediated selection yields cells with altered expression of low-density lipoprotein receptor-related protein. J. Cell Biol..

[35] Holmes B.B., DeVos S.L., Kfoury N., Li M., Jacks R., Yanamandra K., Ouidja M.O., Brodsky F.M., Marasa J., Bagchi D.P., Kotzbauer P.T., Miller T.M., Papy-Garcia D., Diamond M.I. (2013). Heparan sulfate proteoglycans mediate internalization and propagation of specific proteopathic seeds. Proc. Natl. Acad. Sci. U. S. A..

[36] Rauch J.N., Chen J.J., Sorum A.W., Miller G.M., Sharf T., See S.K., Hsieh-Wilson L.C., Kampmann M., Kosik K.S. (2018). Tau internalization is regulated by 6-O sulfation on heparan sulfate proteoglycans (HSPGs). Sci. Rep..

[37] Stopschinski B.E., Holmes B.B., Miller G.M., Manon V.A., Vaquer-Alicea J., Prueitt W.L., Hsieh-Wilson L.C., Diamond M.I. (2018). Specific glycosaminoglycan chain length and sulfation patterns are required for cell uptake of tau versus -synuclein and -amyloid aggregates. J. Biol. Chem..

[38] Esko J.D., Stweart T.E., Taylor W.H. (1985). Animal cell mutants defective in glycosaminoglycan biosynthesis. Proc. Natl. Acad. Sci. U. S. A..

[39] Chappell D.A., Inoue I., Fry G.L., Pladet M.W., Bowen S.L., Iverius P.-H., Lalouel J.-M., Strickland D.K. (1994). Cellular catabolism of normal very low density lipoproteins via the low density lipoprotein receptor-related protein/α2-macroglobulin receptor is induced by the c-terminal domain of lipoprotein lipase. J. Biol. Chem..

[40] Jarosz-Griffiths H.H., Noble E., Rushworth J.V., Hooper N.M. (2016). Amyloid-β receptors: The good, the bad, and the prion protein. J. Biol. Chem..

[41] Nizynski B., Dzwolak W., Nieznanski K. (2017). Amyloidogenesis of tau protein. Protein Sci..

[42] Bramblett G.T., Goedert M., Jakes R., Merrick S.E., Trojanowski J.Q., Lee V.M.Y. (1993). Abnormal tau phosphorylation at Ser396 in Alzheimer’s disease recapitulates development and contributes to reduced microtubule binding. Neuron.

[43] Mandelkow E.M., Biernat J., Drewes G., Gustke N., Trinczek B., Mandelkow E. (1995). Tau domains, phosphorylation, and interactions with microtubules. Neurobiol. Aging.

[44] Hanger D.P., Byers H.L., Wray S., Leung K.Y., Saxton M.J., Seereeram A., Reynolds C.H., Ward M.A., Anderton B.H. (2007). Novel phosphorylation sites in tau from Alzheimer brain support a role for casein kinase 1 in disease pathogenesis. J. Biol. Chem..

[45] Grundke-Iqbal I., Iqbal K., Tung Y.C. (1986). Abnormal phosphorylation of the microtubule-associated protein τ (tau) in Alzheimer cytoskeletal pathology. Proc. Natl. Acad. Sci. U. S. A..

[46] Ihara Y., Nukina N., Miura R., Ogawara M. (1986). Phosphorylated tau protein is integrated into paired helical filaments in Alzheimer’s disease. J. Biochem..

[47] Iqbal K., Grundke-Iqbal I., Smith A.J., George L., Tung Y.C., Zaidi T. (1989). Identification and localization of a τ peptide to paired helical filaments of Alzheimer disease. Proc. Natl. Acad. Sci. U. S. A..

[48] Kounnas M.Z., Chappell D.A., Wong H., Argraves W.S., Strickland D.K. (1995). The cellular internalization and degradation of hepatic lipase is mediated by low density lipoprotein receptor-related protein and requires cell surface proteoglycans. J. Biol. Chem..

[49] Mair W., Muntel J., Tepper K., Tang S., Biernat J., Seeley W.W., Kosik K.S., Mandelkow E., Steen H., Steen J.A. (2016). FLEXITau: Quantifying post-translational modifications of tau protein *in vitro* and in human disease. Anal. Chem..

[50] Tepper K., Biernat J., Kumar S., Wegmann S., Timm T., Hübschmann S., Redecke L., Mandelkow E.M., Müller D.J., Mandelkow E. (2014). Oligomer formation of tau protein hyperphosphorylated in cells. J. Biol. Chem..

[51] Šimić G., Babić Leko M., Wray S., Harrington C., Delalle I., Jovanov-Milošević N., Bažadona D., Buée L., de Silva R., Giovanni G. Di, Wischik C., Hof P.R. (2016). Tau protein hyperphosphorylation and aggregation in Alzheimer’s disease and other tauopathies, and possible neuroprotective strategies. Biomolecules.

[52] Prasad J.M., Young P.A., Strickland D.K. (2016). High affinity binding of the receptor-associated protein D1D2 domains with the low density lipoprotein receptorrelated protein (LRP1) Involves bivalent complex formation: Critical roles of lysines 60 and 191. J. Biol. Chem..

[53] Young P.A., Migliorini M., Strickland D.K. (2016). Evidence that factor VIII forms a bivalent complex with the low density lipoprotein (LDL) receptor-related protein 1 (LRP1): Identification of cluster IV on LRP1 as the major binding site. J. Biol. Chem..

[54] Migliorini M., Li S., Zhou A., Emal C.D., Lawrence D.A., Strickland D.K. (2020). High-affinity binding of plasminogen-activator inhibitor 1 complexes to LDL receptor – related protein 1 requires lysines. J. Biol. Chem..

[55] Neels J.G., Van Den Berg B.M.M., Lookene A., Olivecrona G., Pannekoekt H., Van Zonneveld A.J. (1999). The second and fourth cluster of class A cysteine-rich repeats of the low density lipoprotein receptor-related protein share ligand-binding properties. J. Biol. Chem..

[56] Obermoeller-McCormick L.M., Li Y., Osaka H., FitzGerald D.J., Schwartz A.L., Bu G. (2001). Dissection of receptor folding and ligand-binding property with functional minireceptors of LDL receptor-related protein. J. Cell Sci..

[57] Ruiz J., Kouiavskaia D., Migliorini M., Robinson S., Saenko E.L., Gorlatova N., Li D., Lawrence D., Hyman B.T., Weisgraber K.H., Strickland D.K. (2005). The apoE isoform binding properties of the VLDL receptor reveal marked differences from LRP and the LDL receptor. J. Lipid Res..

[58] Arai A.L., Migliorini M., Au D.T., Hanh-dantona E., Stetler-stevenson W.G., Muratoglu S.C., Strickland D.K. (2020). High-affinity binding of LDL receptor – related protein 1 to matrix metalloprotease 1 requires protease: Inhibitor complex formation. Biochemistry.

[59] Holmes B.B., Furman J.L., Mahan T.E., Yamasaki T.R., Mirbaha H., Eades W.C., Belaygorod L., Cairns N.J., Holtzman D.M., Diamond M.I. (2014). Proteopathic tau seeding predicts tauopathy *in vivo*. Proc. Natl. Acad. Sci. U. S. A..

[60] Evans L., Strano A., Campbell A., Karakoc E., Iorio F., Bassett A., Livesey F. (2020). Whole genome CRISPR screens identify LRRK2-regulated endocytosis as a major mechanism for extracellular tau uptake by human neuorns. bioRxiv.

[61] Kounnas M.Z., Morris R., Thompson M.R., FitzGerald D.J., Strickland D.K., Saelinger C.B. (1992). The alpha-macroglobulin receptor/LDL receptor-related protein binds and internalizes Pseudomonas exotoxin A. J. Biol. Chem..

[62] Zdanovsky A.G., Zdanovskaia M.V., Strickland D., FitzGerald D.J. (1996). Ligand-toxin hybrids directed to the α2-macroglobulin receptor/low density lipoprotein receptor-related protein exhibit lower toxicity than native Pseudomonas exotoxin. J. Biol. Chem..

[63] Mahley R.W., Innerarity T.L., Pitas R.E., Weisgraber K.H., Brown J.H., Gross E. (1977). Inhibition of lipoprotein binding to cell surface receptors of fibroblasts following selective modification of arginyl residues in arginine rich and B apoproteins. J. Biol. Chem..

[64] Weisgraber K.H., Innerarity T.L., Mahley R.W. (1978). Role of the lysine residues of plasma lipoproteins in high affinity binding to cell surface receptors on human fibroblasts. J. Biol. Chem..

[65] Arandjelovic S., Hall B.D., Gonias S.L. (2005). Mutation of lysine 1370 in full-length human α2- macroglobulin blocks binding to the low density lipoprotein receptor-related protein-1. Arch. Biochem. Biophys..

[66] Migliorini M.M., Behre E.H., Brew S., Ingham K.C., Strickland D.K. (2003). Allosteric modulation of ligand binding to low density lipoprotein receptor-related protein by the receptor-associated protein requires critical lysine residues within its carboxyl-terminal domain. J. Biol. Chem..

[67] Fisher C., Beglova N., Blacklow S.C. (2006). Structure of an LDLR-RAP complex reveals a general mode for ligand recognition by lipoprotein receptors. Mol. Cell.

[68] Dolmer K., Campos A., Gettins P.G.W. (2013). Quantitative dissection of the binding contributions of ligand lysines of the receptor-associated protein (RAP) to the low density lipoprotein receptor-related protein (LRP1). J. Biol. Chem..

[69] Fitzpatrick A.W.P., Falcon B., He S., Murzin A.G., Murshudov G., Garringer H.J., Crowther R.A., Ghetti B., Goedert M., Scheres S.H.W. (2017). Cryo-EM structures of tau filaments from Alzheimer’s disease. Nature.

[70] Willard L., Ranjan A., Zhang H., Monzavi H., Boyko R.F., Sykes B.D., Wishart D.S. (2003). Vadar: A web server for quantitative evaluation of protein structure quality. Nucleic Acids Res..

[71] Prasad H., Rao R. (2018). Amyloid clearance defect in ApoE4 astrocytes is reversed by epigenetic correction of endosomal pH. Proc. Natl. Acad. Sci. U. S. A..

[72] Chen Y., Durakoglugil M.S., Xian X., Herz J. (2010). ApoE4 reduces glutamate receptor function and synaptic plasticity by selectively impairing ApoE receptor recycling. Proc. Natl. Acad. Sci. U. S. A..

[73] Shi Y., Yamada K., Liddelow S.A., Smith S.T., Zhao L., Luo W., Tsai R.M., Spina S., Grinberg L.T., Rojas J.C., Gallardo G., Wang K., Roh J., Robinson G., Finn M.B. (2017). ApoE4 markedly exacerbates tau-mediated neurodegeneration in a mouse model of tauopathy. Nature.

[74] Arboleda-Velasquez J.F., Lopera F., O’Hare M., Delgado-Tirado S., Marino C., Chmielewska N., Saez-Torres K.L., Amarnani D., Schultz A.P., Sperling R.A., Leyton-Cifuentes D., Chen K., Baena A., Aguillon D., Rios-Romenets S. (2019). Resistance to autosomal dominant Alzheimer’s disease in an APOE3 christchurch homozygote: A case report. Nat. Med..

[75] Lalazar A., Weisgraber K.H., Rall S.C., Giladi H., Innerarity T.L., Levanon A.Z., Boyles J.K., Amit B., Gorecki M., Mahley R.W., Vogel T. (1988). Site-specific mutagenesis of human apolipoprotein E. Receptor binding activity of variants with single amino acid substitutions. J. Biol. Chem..

[76] Dujardin S., Commins C., Lathuiliere A., Beerepoot P., Fernandes A.R., Kamath T.V., De Los Santos M.B., Klickstein N., Corjuc D.L., Corjuc B.T., Dooley P.M., Viode A., Oakley D.H., Moore B.D., Mullin K. (2020). Tau molecular diversity contributes to clinical heterogeneity in Alzheimer’s disease. Nat. Med..

[77] Beisiegel U., Weber W., Ihrke G., Herz J., Stanley K.K. (1989). The LDL-receptor-related protein, LRP, is an apolipoprotein E-binding protein. Nature.

[78] Kowal R.C., Herz J., Goldstein J.L., Esser V., Brown M.S. (1989). Low density lipoprotein receptor-related protein mediates uptake of cholesteryl esters derived from apoprotein E-enriched lipoproteins. Proc. Natl. Acad. Sci. U. S. A..

[79] Ashcom J.D., Tiller S.E., Dickerson K., Cravens J.L., Argraves W.S., Strickland D.K. (1990). The human alpha 2-macroglobulin receptor: Identification of a 420-kD cell surface glycoprotein specific for the activated conformation of alpha 2-macroglobulin. J. Cell Biol..

[80] Williams S.E., Ashcom J.D., Argraves W.S., Strickland D.K. (1992). A novel mechanism for controlling the activity of alpha 2-macroglobulin receptor/low density lipoprotein receptor-related protein. Multiple regulatory sites for 39-kDa receptor-associated protein. J. Biol. Chem..

[81] Strickland D.K., Ashcom J.D., Williams S., Burgess W.H., Migliorini M., Argraves W.S. (1990). Sequence identity between the alpha 2-macroglobulin receptor and low density lipoprotein receptor-related protein suggests that this molecule is a multifunctional receptor. J. Biol. Chem..

[82] Furman J.L., Holmes B.B., Diamond M.I. (2015). Sensitive detection of proteopathic seeding activity with FRET flow cytometry. J. Vis. Exp..

[83] Rueden C.T., Schindelin J., Hiner M.C. (2017). ImageJ2: ImageJ for the next generation of scientific image data. BMC Bioinform.

